# Potassium supplementation and depletion during development of salt-sensitive hypertension in male and female SS rats

**DOI:** 10.1172/jci.insight.181778

**Published:** 2025-04-15

**Authors:** Adrian Zietara, Lashodya V. Dissanayake, Melissa Lowe, Biyang Xu, Vladislav Levchenko, Vasundhara Kain, Ganesh V. Halade, Christine A. Klemens, Oleg Palygin, Alexander Staruschenko

**Affiliations:** 1 Department of Molecular Pharmacology and Physiology,; 2 Department of Internal Medicine, and; 3 Hypertension and Kidney Research Center, University of South Florida, Tampa, Florida, USA.; 4 Department of Medicine, Division of Nephrology, Medical University of South Carolina, Charleston, South Carolina, USA.; 5 James A. Haley Veterans Hospital, Tampa, Florida, USA.

**Keywords:** Cell biology, Nephrology, Chronic kidney disease, Hypertension, Potassium channels

## Abstract

The dietary sodium/potassium ratio is positively correlated with blood pressure, and understanding this relationship is crucial for improving hypertension treatment. Moreover, few studies have examined these effects in both sexes. In this study, we aimed to investigate how supplementing (1.41% K^+^; HK) or depleting (DK) dietary potassium affects the development of salt-sensitive (SS) hypertension in male and female Dahl SS rats. Potassium supplementation attenuated blood pressure during 5 weeks of high-salt (4% NaCl) diet in male but not in female rats. In contrast, a potassium-deficient diet prevented the development of salt-induced hypertension in both sexes, though this effect is unlikely to be protective. Both males and females on the DK diet were hypokalemic and had diminished heart rates and reduced weight gain; furthermore, females experienced high mortality. RNA-Seq of kidney cortical tissue revealed a number of genes that may underlie the sex-specific differences in phenotype. Male rats supplemented with potassium exhibited a decreased number and size of WNK4 puncta, whereas in potassium-supplemented females, there was no difference in puncta count and there was an increase in puncta size. Our data indicate there are sex-dependent differences in response to dietary potassium in hypertension and that the distal nephron compensates for severe potassium deficiency.

## Abstract 2

Dietary potassium affects blood pressure differently in males and females, highlighting the importance of sex-specific responses in salt-sensitive hypertension and kidney function.

## Graphical abstract

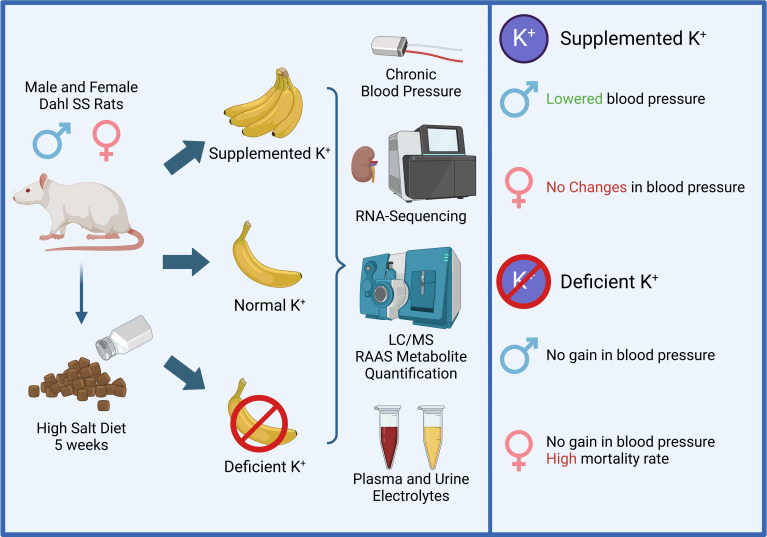

## Introduction

Sodium is a significant factor in blood pressure regulation, where increased sodium intake can result in a profound elevation of blood pressure. Due to the prevalence of sodium in the modern Western diet, the rates of hypertension, cardiovascular disease (CVD), and other comorbidities have been steadily increasing; under the revised guidelines from the American Heart Association (AHA) and other organizations, nearly 50% of adults in the United States are classified as hypertensive (1–3). It was estimated that reducing daily sodium intake by 3 g would dramatically reduce rates of CVD and resulting deaths (4). Many commonly prescribed antihypertensive drugs function by increasing the net excretion of sodium. However, despite the availability of these treatments, many patients still struggle to maintain their blood pressure at a desired level (3, 5–7).

Opposite to sodium, intake of potassium is correlated with a decrease in blood pressure, which was first observed about a century ago (8–10). After some time, it was discovered that the molar Na^+^/K^+^ ratio is a prominent factor in the severity of hypertension development (11). In recent years, interest has been garnered in potassium’s potential for alleviating high blood pressure. Multiple recent epidemiological studies have studied the role of potassium in blood pressure; the Prospective Urban Rural Epidemiology (PURE) study examined more than 100,000 adults from around the world and reported that potassium excretion inversely correlated with systolic blood pressure; follow-up of about several years associated increased potassium excretion to decreased risk of major cardiovascular events and death (12–14). A clinical trial utilizing the Dietary Approaches to Stop Hypertension (DASH) diet observed that the diet, which includes many fruits and vegetables and is high in potassium, lowered blood pressure of patients, even those consuming high amounts of sodium (15, 16). Another study conducted in rural China observed that the substitution of sodium chloride with 75% sodium chloride and 25% potassium chloride significantly lowered the risk of stroke, major cardiovascular events, and death in people aged 60 years old or higher with high blood pressure (17).

Previous work has discovered various mechanisms that describe how potassium may lower blood pressure. One of the areas in which potassium has been shown to be beneficial is in vasculature (18, 19). It was shown that increased extracellular potassium activated inward rectifying potassium (K_ir_) channels in vascular smooth muscle cells, causing hyperpolarization and relaxation of the muscle cells (20, 21). Also, it was suggested that high potassium may be involved in endothelium-derived hyperpolarizing factor–mediated responses, which can cause vasodilation through endothelium-dependent relaxation (22–24). A high-potassium diet was also shown to be protective against vascular damage through suppression of reactive oxygen species (ROS) production (25). Additionally, potassium supplementation has been well established to yield beneficial effects in the kidney. High potassium intake has been shown to induce natriuresis (26, 27). Some evidence has shown that increased potassium load reduced sodium reabsorption in the proximal tubule and the loop of Henle (28, 29). Multiple studies have shown that high potassium loading plays a large role in the distal convoluted tubule (DCT), where increased potassium dephosphorylates the sodium chloride cotransporter (NCC), resulting in inhibition of active NCC and decreased sodium reabsorption (27, 30–33). Potassium also plays a role in renoprotection; high dietary potassium was shown to decrease albuminuria, glomerular and tubulointerstitial damage, and renal lesions (34–38).

The study of potassium’s effects on blood pressure in animal models has been conducted for many decades, though to the best of our knowledge, it has not been thoroughly studied in the salt-sensitive (SS) rat, a translatable low renin model of salt sensitivity (39–41). Furthermore, little, if any, previous work examined females, which, in other models, have been shown to exhibit differences in electrolyte transport compared with males (42–44). Here, we utilize a high-NaCl diet with deficient potassium (DK), normal potassium (NK), and high potassium (HK) content to determine how the kidneys of both male and female rats adapt and compensate for these conditions during the development of SS hypertension.

## Results

### Effects of potassium on the development of SS hypertension and survival.

**Figure 1:**
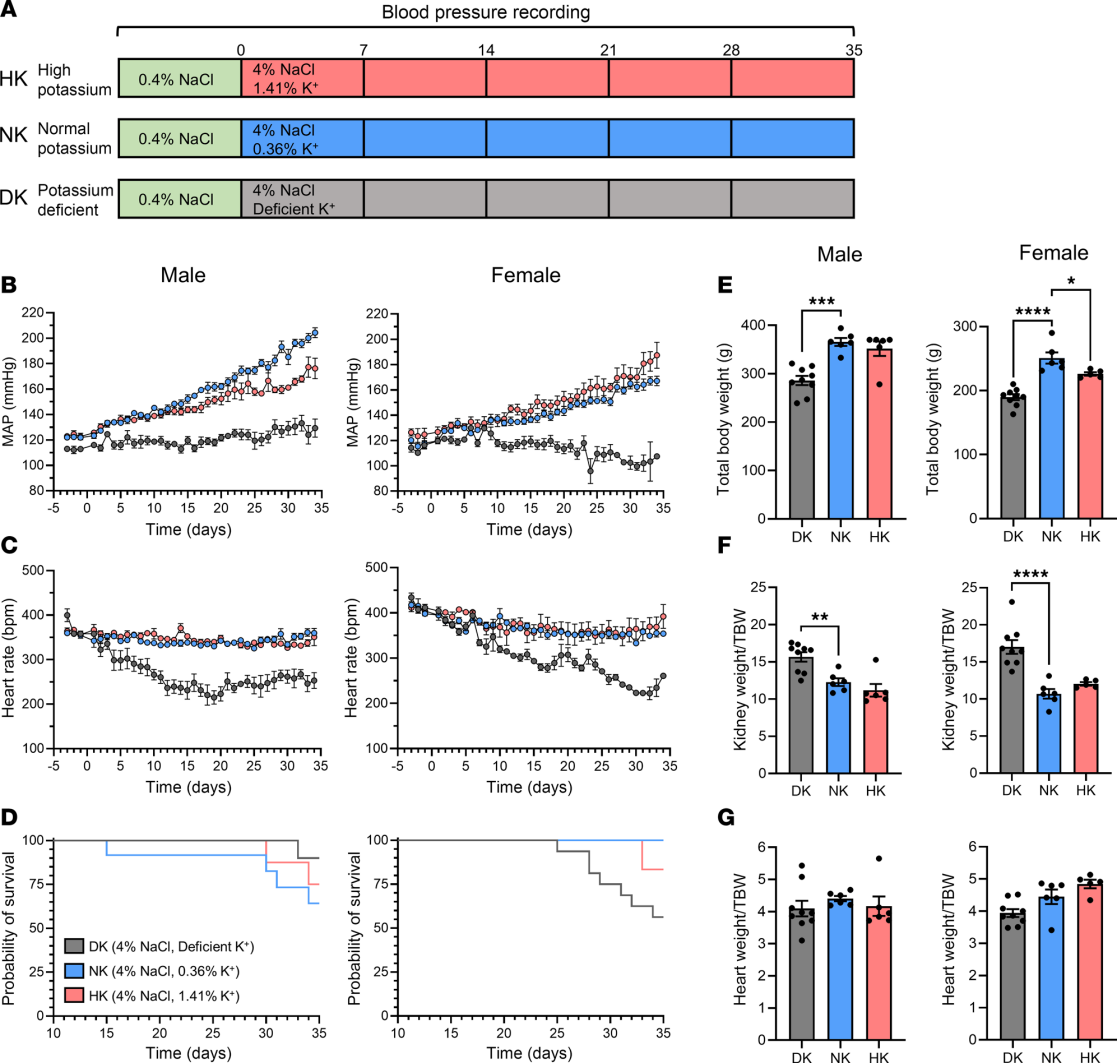
Potassium deficiency significantly attenuates the development of SS hypertension in male and female rats. (**A**) Diagram of the experimental protocol. (**B** and **C**) Daily average mean arterial pressure (MAP) and heart rate of male and female SS rats during 5 weeks on a 4% NaCl diet with either deficient (DK), normal (NK), or high (HK) potassium. Daily values are averages of recordings from 9:00 a.m. to 12:00 p.m. Two-way repeated-measures ANOVA was used to compare differences between groups. Data are shown as mean ± SEM, *n* ≥ 5 for males and *n* ≥ 5 for females (*n* ≥ 1 for female DK group). (**D**) Kaplan-Meier survival probability of male and female rats on high salt diets. For male rats: DK (*n* = 11), NK (*n* = 12), and HK (*n* = 8). For female rats: DK (*n* = 16), NK (*n* = 6), and HK (*n* = 6). (**E**) Endpoint total body weights (TBW). (**F**) Endpoint kidney weight normalized to TBW. (**G**) Endpoint heart weight normalized to TBW. One-way ANOVA was used to compare differences compared with the control (NK). Data are shown as mean ± SEM; *n* ≥ 6 for males and *n* ≥ 5 for females. *P < 0.05, **P < 0.01, ***P < 0.001, ****P < 0.0001.

Previously, we observed that potassium supplementation does not attenuate the development of SS hypertension until after 3 weeks of diet, with a profound attenuation by week 5; thus, we utilized a 5-week dietary protocol (Figure 1A) (45). Over the course of the 5-week high-salt (4% NaCl) diet, male and female animals fed the NK diet (0.36% K^+^) developed high blood pressure (Figure 1B), which was attenuated in the potassium-supplemented HK (1.41% K^+^) males but not females and was even further reduced in the DK rats; endpoint mean arterial pressure (MAP) and heart rates are shown in Supplemental Figure 1, A and B (supplemental material available online with this article; https://doi.org/10.1172/jci.insight.181778DS1). Systolic and diastolic blood pressures followed similar trends (Supplemental Figure 2, A and B). Once the dietary protocols commenced, heart rates began progressively decreasing in the DK rats (Figure 1C), while the NK and HK group heart rates remained relatively constant over the 5 weeks. As the blood pressure continued to rise on the high-salt diets, some rats began spontaneously dying or, in some cases, suffered strokes and had to be euthanized early. For male rats, survival probability (Figure 1D) correlated with MAP, whereas NK rats had the lowest survival, followed by HK rats. Surprisingly, DK rats experienced the best survival outcomes. Female HK and NK rats experienced improved survival compared with their male counterparts; however, in contrast to males, the female DK group had the highest mortality rate out of any of the groups. At the endpoint, rats were weighed, which revealed that the total body weight was reduced (Figure 1E) in male and female DK rats and slightly decreased in female HKs. The kidney/bodyweight ratio (Figure 1F) was significantly higher in male and female DK rats, likely due to their lower body weight; however, heart/bodyweight ratios (Figure 1G) were not different between the groups. Kidney size was likely unaffected by the diet and resulting phenotype, but the heart and skeletomuscular development seem to have been impaired.

### Effects of dietary potassium on kidney and cardiac injury and on electrolyte homeostasis.

**Figure 2:**
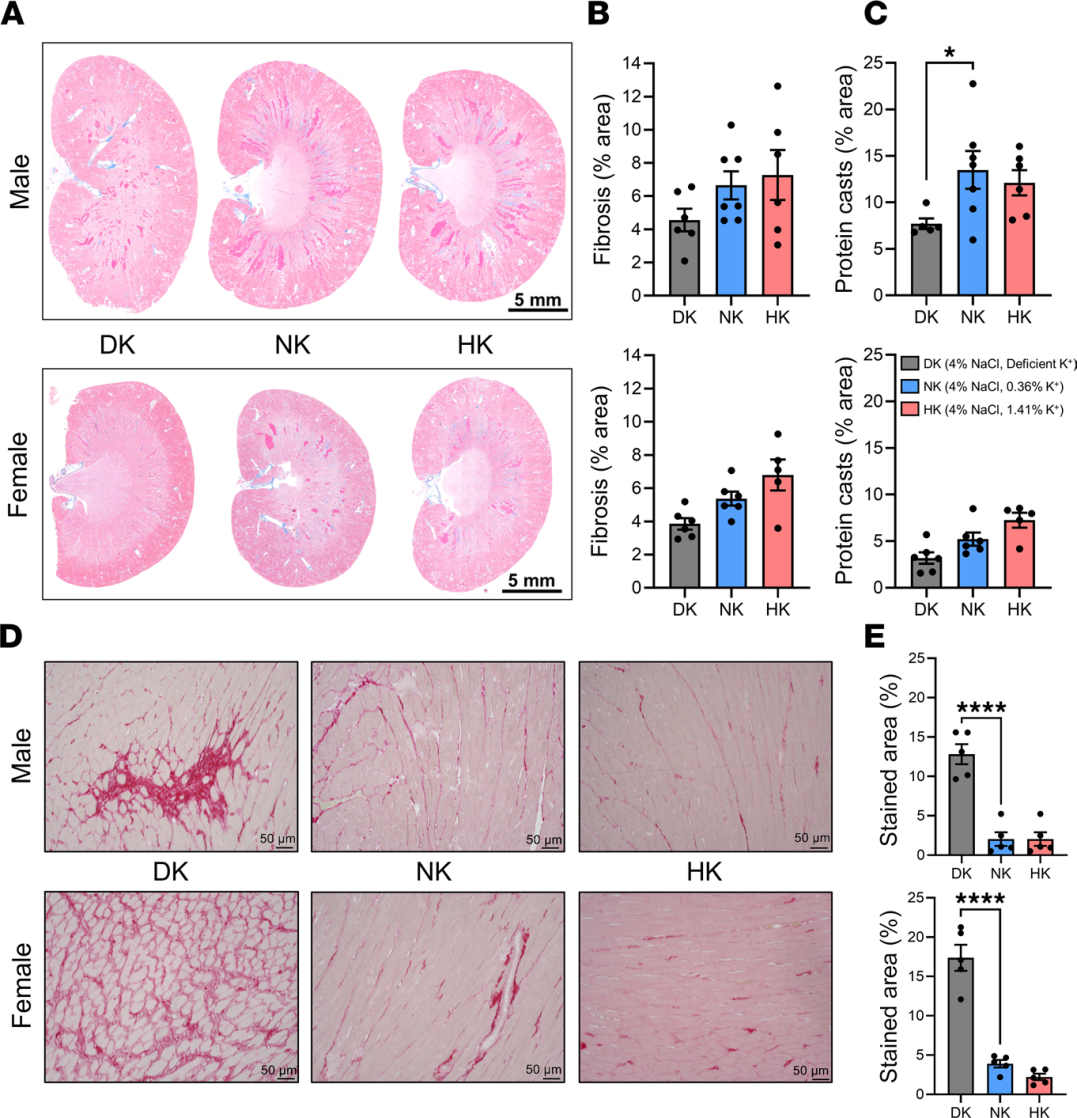
Effects of dietary potassium on renal and cardiac damage in SS hypertension. (**A**) Representative images of whole kidneys stained with Masson’s trichrome from male and female rats on DK, NK, and HK diets. Scale bars: 5 mm. (**B**) Percentage of cortical fibrosis. (**C**) Percentage of medullary protein casts. (**D**) Representative 20× images of picrosirius red staining in left ventricles from male and female rats on DK, NK, and HK diets. Scale bars: 50 μm. (**E**) Collagen percentage of stained area in the left ventricles of DK, NK, and HK rats. One-way ANOVA was used to compare differences compared with the control (NK). Data are shown as mean ± SEM; *n* ≥ 5 for males and females. *P < 0.05, ****P < 0.0001.

Next, we examined how the different dietary conditions affected renal damage and electrolyte homeostasis. The kidney is vital for maintaining electrolyte homeostasis, so we examined the extent of renal damage after 5 weeks of the high-salt diet. Masson’s trichrome staining of kidney tissue (Figure 2A) revealed no differences in cortical fibrosis (Figure 2B), and only male DK rats displayed significantly reduced medullary protein casts (Figure 2C); however, we did not observe any renoprotection in potassium-supplemented animals. Picrosirius red (PSR) staining was performed in heart tissue; representative images are shown in Figure 2D. Quantification of PSR staining showed extensive collagen remodeling in the heart tissue of DK male and female rats (Figure 2E), which is consistent with profound HR changes shown in Figure 1C.

Analysis of endpoint plasma electrolytes revealed that male and female DK rats became severely hypokalemic (Table 1) after 5 weeks of the diet, and the HK groups experienced elevated plasma K^+^. There were no differences in plasma Na^+^ in male rats, but in the female group, the DK rats exhibited elevated plasma Na^+^. Plasma Ca^2+^ was decreased in the male and female DK group, plasma Cl^–^ was unchanged for all groups, and plasma creatinine was significantly lower only in DK males, likely due to reduced muscle mass. The day before the endpoint, animals were placed in metabolic cages for 24-hour urine collection. There were no differences in 24-hour urine volume (Table 2). Twenty-four–hour kaliuria correlated with the potassium contents of the diets. It was significantly decreased in DK rats and increased in HK rats, while natriuresis was only significantly decreased in the DK group. Female DK rats excreted significantly more Cl^–^, and male DK rats excreted more creatinine.

### Analysis of plasma RAAS metabolites.

**Figure 3:**
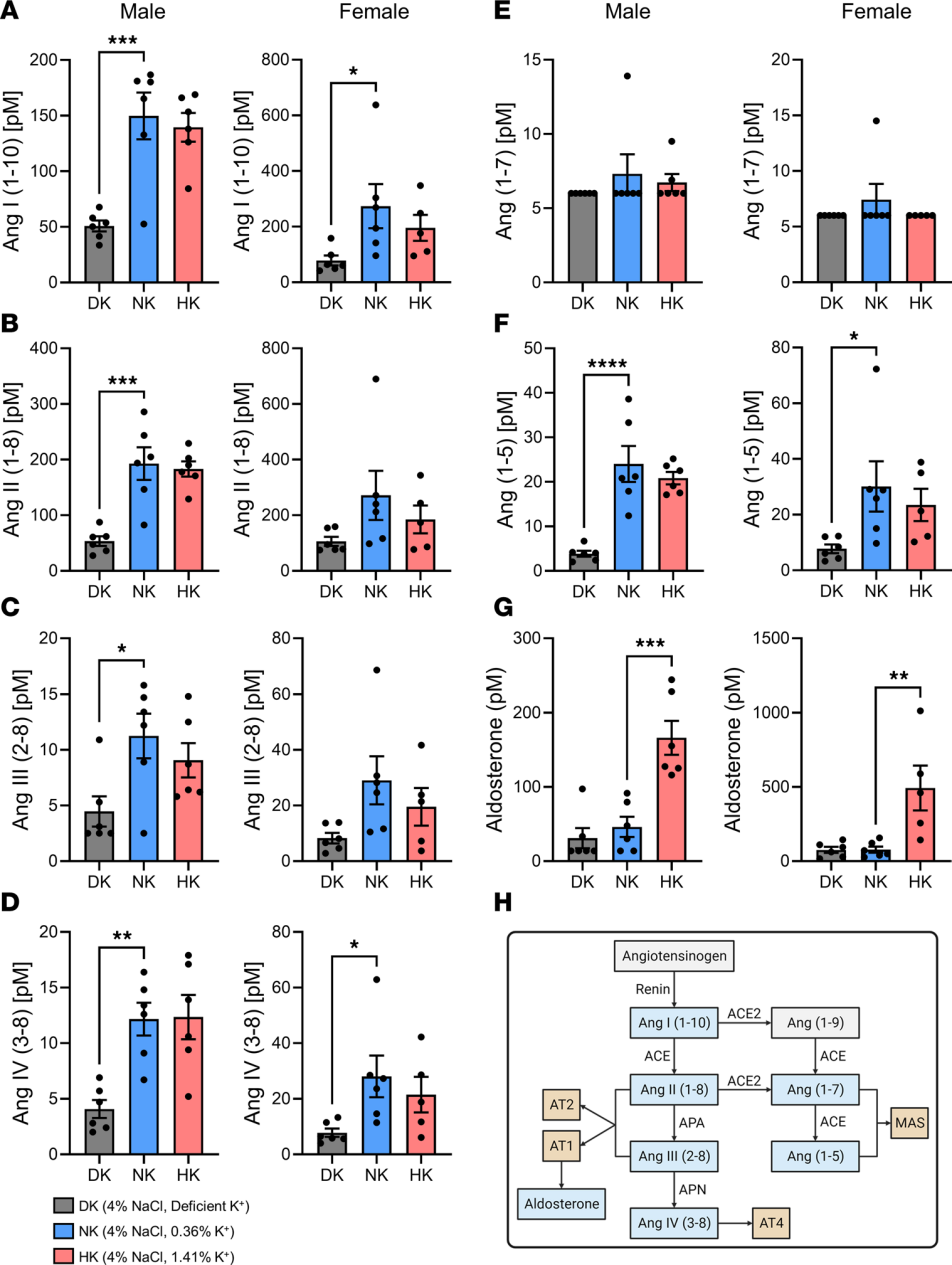
Quantification of plasma RAAS metabolites. (**A**–**G**) Circulating plasma Ang I (1–10) (**A**), Ang II (1–8) (**B**), Ang III (2–8) (**C**), Ang IV (3–8) (**D**), Ang (1–7) (**E**), Ang (1–5) (**F**), and aldosterone (**G**). One-way ANOVA was used to compare differences compared with the control (NK). Data are shown as mean ± SEM; *n* ≥ 6 for males and *n* ≥ 5 for females. (**H**) Diagram of RAAS pathway. *P < 0.05, **P < 0.01, ***P < 0.001, ****P < 0.0001.

It is well known that the renin-angiotensin-aldosterone system (RAAS) plays a significant role in potassium homeostasis and that potassium levels can influence RAAS, which we have previously shown in *Kcnj16*-KO SS rats (46, 47). Accordingly, it was pertinent to measure how the different diets affected RAAS in these experiments. Stimulating RAAS can increase the secretion of potassium; expectedly, many of the RAAS metabolites (Figure 3H) were significantly attenuated in the DK groups; however, most of the studied metabolites were unchanged in the HK groups. In DK males, Ang I (1–10), Ang II (1–8), Ang III (2–8), and Ang IV (3–8) (Figure 3, A–D) were significantly decreased, while in DK females only Ang I (1–10) and Ang IV (3–8) were significantly lower. Examining components of the alternate RAS axis, which primarily acts by increasing nitric oxide and prostaglandins, revealed no differences in Ang (1–7) for either males or females (Figure 3E), but Ang (1–5) was significantly decreased in DK males (Figure 3F) (48). Lastly, aldosterone, which is well documented in responding to dietary changes in potassium, was significantly augmented in male and female HK animals (Figure 3G). Male and female values are compared separately in Supplemental Figure 3. The AA2 ratio (aldosterone/Ang II), which is a measure of the renin-dependent production of aldosterone, was significantly increased in HK females (Supplemental Figure 4A). Plasma renin activity (estimated PRA [PRA-S] [Ang I + Ang II]) is an indication of the overall activation of RAAS and was significantly lower only in DK males (Supplemental Figure 4B). Lastly, ACE activity (ACE-S [Ang II/Ang I]), an estimation of the formation of Ang II from Ang I, was not different between groups except for DK females (Supplemental Figure 4C).

### Changes in the transcriptome are predicted to affect hypertension and ion transport.

We performed bulk RNA-Seq analyses of the kidney cortices of all groups to obtain a broader understanding of the mechanisms involved. First, a few common practices were used to make sure our data were grouped appropriately. Hierarchical clustering of the samples based upon which genes are differentially expressed (DE) (Supplemental Figure 5A) indicates that, when compared with their respective NK controls, male and female DK rats experienced less homogeneity in gene expression compared with the HK groups. A PCA plot (Supplemental Figure 5B) of the data sets illustrates the similarity of expression profiles between NK and HK groups and distinct deviation in the male and female DK groups. Consequently, functional analyses were done using ingenuity pathway analysis (IPA) software.

In total, 3,986 and 1,718 genes were, respectively, DE (based on only *P* value cut-off) compared with NK in males. Based on a combined score of *P* value and fold change, the top 20 DE genes are highlighted in Supplemental Figure 6, A and B. Among the top disease and function annotations, “transport of molecules” was upregulated and “ion homeostasis of cells” was downregulated in both DK and HK groups compared with NK (Supplemental Figure 6C). Male DK versus NK comparison had a more robust annotation list than HK versus NK with several Ca^2+^ handling functions such as “Flux of Ca^2+^” and “Quantity of Ca^2+^” being enhanced. “Quantity of ROS”, “Fibrosis”, and “Synthesis of NO” were some of the other compelling annotations predicted in DK male rats to be affected. Interestingly, many of the top canonical pathways in both DK and HK versus NK were related to calcium signaling (GABAergic, Orexin, CREB signaling, etc.) (Supplemental Figure 6D).

Exploring the overall transcriptomic differences of the female groups, 6,015 and 4,667 DE genes were noted based on *P* value alone (Supplemental Figure 7, A and B). While “transport of molecules”, “inflammatory response”, and “vasculogenesis” were among the common annotations in both DK and HK female groups, “excretion of Na^+^”, “blood pressure”, and “quantity of Ca^2+^” were among the unique annotations in the DK group (Supplemental Figure 7C). Several inflammatory pathways (IL-4, IL-33, cytokine storm signaling, etc.) and redox signaling pathways (NRF2-mediated oxidative stress response, glutathione redox reaction, etc.) were enhanced in DK versus NK females (Supplemental Figure 7D).

**Figure 4:**
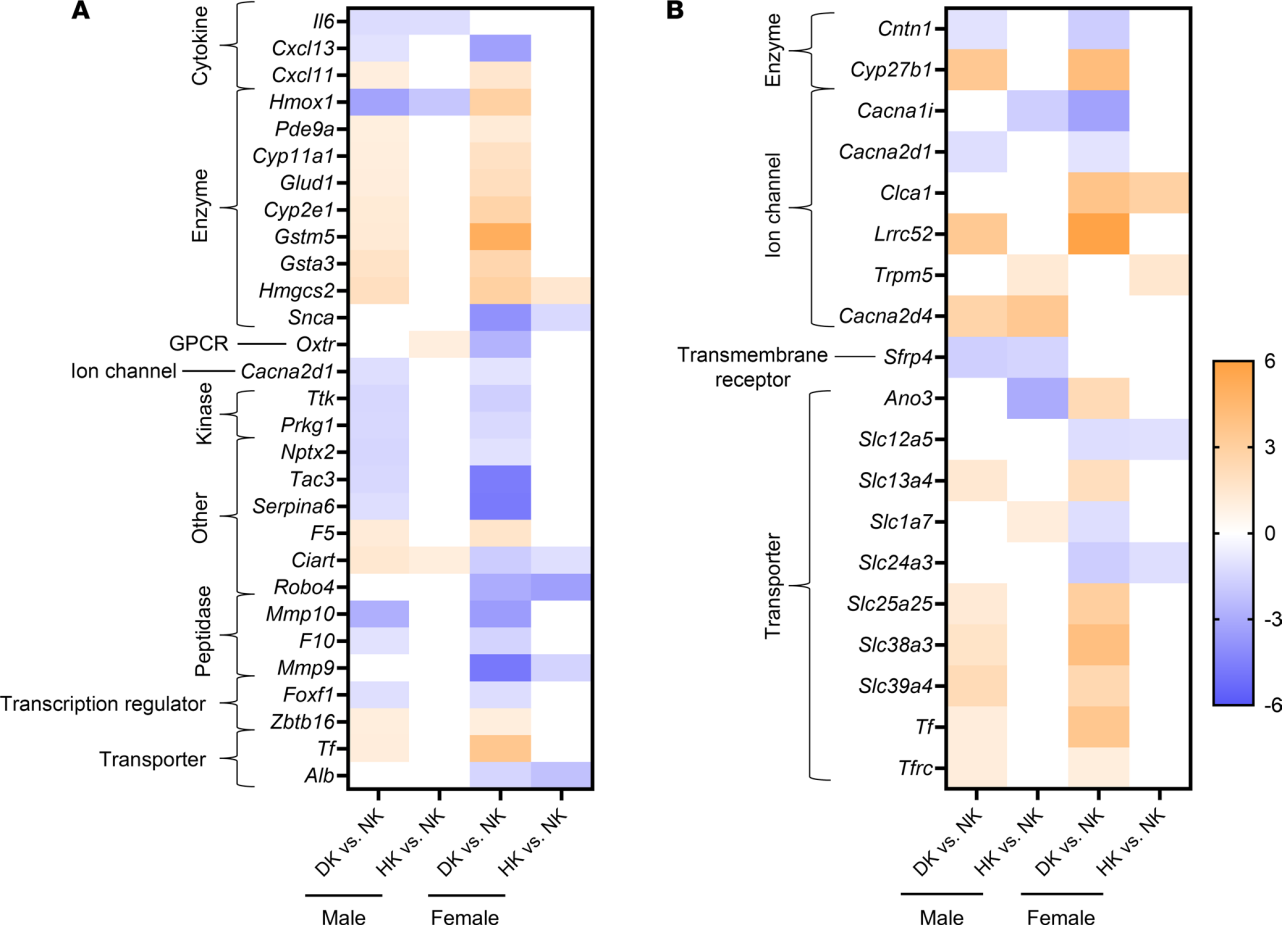
Hypertension and ion transport related differentially expressed (DE) genes. (**A** and **B**) Hypertension-related DE genes (**A**) and ion-transport-related DE genes (**B**) in DK and HK groups each compared with the respective NK within a sex. Only the genes that are at least changing in 2 groups are shown. Full lists can be found in Supplemental Tables 1–8. Only genes that are ≥ 1 or ≤ –1 in fold change and < 0.05 in *P* value were considered DE. *P* value was calculated using the Wald test.

We mapped all DE genes for different comparisons with the IPA knowledge base to highlight the genes that are related to the terms “hypertension” and “ion transport” (Supplemental Tables 1–8). Any genes that are DE in more than 1 comparison are highlighted in Figure 4, A and B. Both male and female rats had a lower progression of hypertension in DK groups. Out of the “hypertension” and “ion transport” related genes, 11 were downregulated (*Cntn1*, *Cacna2d1*, *Cxcl13*, *Ttk*, *Prkg1*, *Nptx2*, *Tac3*, *Serpina6*, *Mmp10*, *F10*, and *Foxf1*) and 19 were upregulated (*Cyp27b1*, *Lrrc52*, *Slc13a4*, *Slc25a25*, *Slc38a3*, *Slc39a4*, *Tf*, *Tfrc*, *Cxcl11*, *Pde9a*, *Cyp11a1*, *Glud1*, *Cyp2e1*, *Gstm5*, *Gsta3*, *Hmgcs2*, *Zbtb16*, *F5*, and *Tf*) commonly in both DK groups (Figure 4, A and B). Although both male and female DK groups had similar blood pressure, the male DK group exhibited better survival rates than their female counterparts. Therefore, it is worth noting that some of the genes were uniquely DE in each of these groups. DK males upregulated *Calb1*, *Klk1*, and *Kcnk4*, while DK females upregulated *Edn1*, *Egfr*, *Ren*, *Nppa*, compared with their respective NK groups (Supplemental Tables 1, 2, 5, and 6). Some notable genes that were only downregulated in female DKs were *Kl*, *Nos1*, and *Pcsk6*.

Although at different levels, male DK and HK both had lower blood pressure at the endpoint. In genes related to “hypertension,” 3 genes were commonly changing in both male DK and HK groups (*Il6*, *Hmox1* downregulated and *Ciart* upregulated) (Figure 4A). Interestingly, *Ciart* was downregulated in both DK and HK female groups compared with NK.

**Figure 5:**
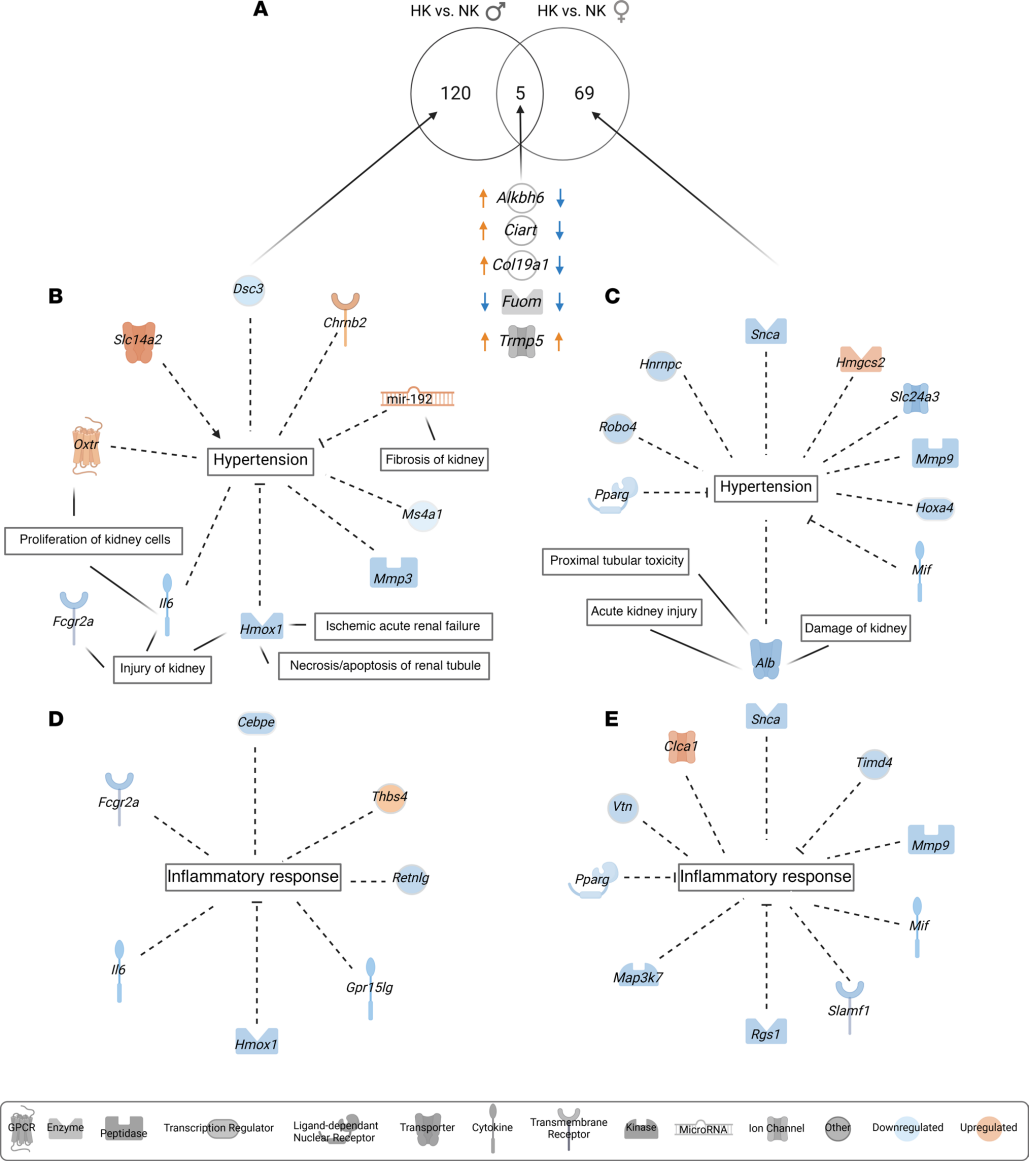
Candidate genes for the phenotype difference between males and females in response to HK diet. (**A**) Venn diagram showing the DE genes for HK versus NK within each respective sex. Five genes were commonly DE and are shown. (**B**) Hypertension and kidney injury–related genes from the uniquely DE genes in male HK versus NK. (**C**) Hypertension and kidney injury–related genes from the uniquely DE genes in female HK versus NK. (**D**) Inflammatory response–related genes from the uniquely DE genes in male HK versus NK. (**E**) Inflammatory response–related genes from the uniquely DE genes in female HK versus NK. Only genes that are ≥ 1 or ≤ –1 in fold change and < 0.05 in *P* value were considered DE. *P* value was calculated using the Wald test.

We observed that, while male rats had an attenuation of hypertension with the HK diet, females did not. To tease apart the possible mechanisms behind this, we explored the DE genes that are common and unique for each sex in the HK-diet challenge (Figure 5, A–C). In total, 125 and 74 genes were DE (*P* < 0.05 and fold change ≥ 1 or ≤ –1) in HK versus NK comparisons in male and female groups in turn (Figure 5A). Five genes were commonly expressed, with 3 of them (*Alkbh6*, *Ciart*, and *Col19a1*) upregulated in HK-fed males while downregulated in the female HK group. Out of the unique DE genes within each sex, we highlighted the ones that are mapped to the term “hypertension” as well as kidney injury-related terms (Figure 5, B and C). *Il6* and miR-192, which are known to be associated with SS hypertension, were among them for the male group (Figure 5B) (49, 50). miR-192, whose upregulation should inhibit hypertension, is also known to affect fibrosis in the kidney (49, 51). In the female HK group but not the males, *Alb* (albumin), which is known to be a robust marker of kidney health, was downregulated (Figure 5C) (52). *Pparg* is thought to be protective against hypertension and was another gene that was downregulated in HK-fed females (53). *Slc24A3* encoding for potassium-dependent sodium/calcium exchanger 3 (Nckx3) was downregulated in females who were not responsive to HK. This gene has been linked to SS hypertension in a human genome-wide study (54). Increased levels of aldosterone can cause inflammation through stimulation of ROS, and due to the observed increase of aldosterone in the HK groups, we also examined genes mapped to “inflammation” (55). Between males and females, there were no commonly expressed inflammation-associated genes. In males, the gene encoding thrombospondin-4, *Thbs4*, was upregulated (Figure 5D), while in females, the only upregulated gene was *Clca1* (Figure 5E), which encodes calcium-activated chloride channel regulator 1.

### Changes in the protein expression of channels and transporters.

**Figure 6:**
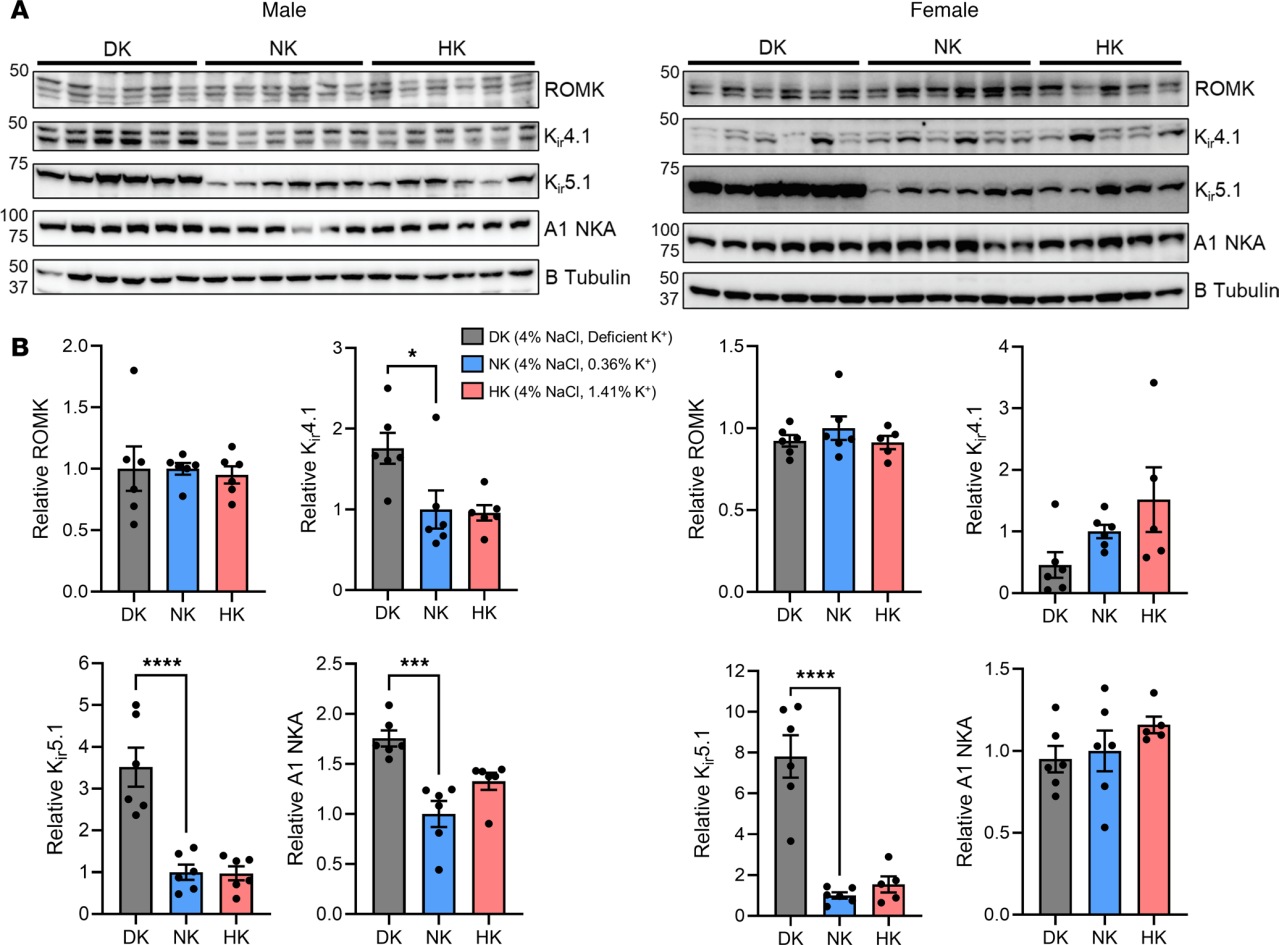
Changes in expression of channels and transporters following a HS diet. (**A**) Western blotting analysis of ROMK, K_ir_4.1, K_ir_5.1, and ANKA subunit in male and female Dahl SS rats. (**B**) Summary of relative densitometry. Data are shown as mean ± SEM; *n* = 6 for males and *n* ≥ 5 for females. One-way ANOVA was used to compare differences compared with the control (NK). *P < 0.05,***P < 0.001, ****P < 0.0001.

**Figure 7:**
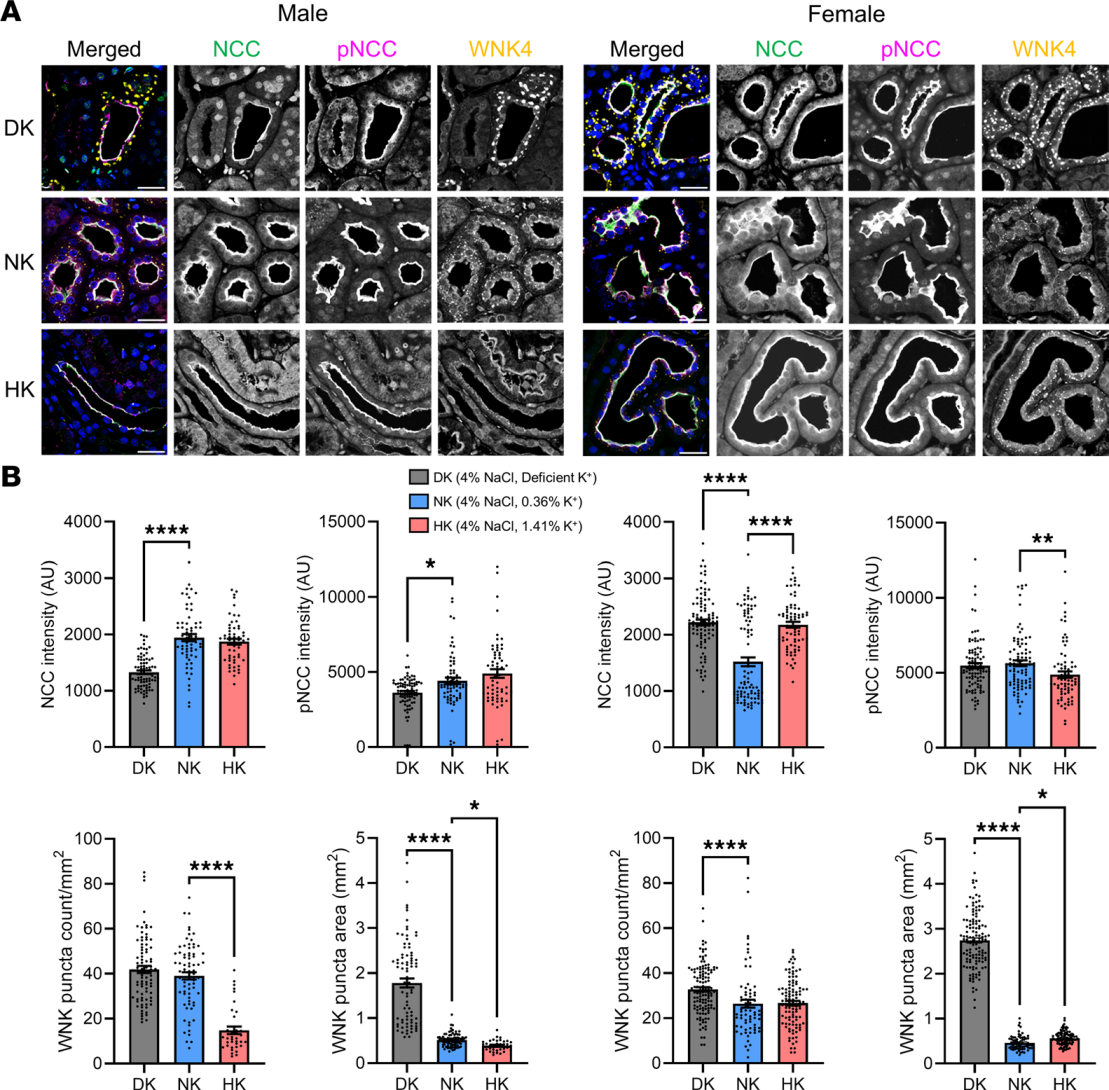
Males and females exhibit altered adaptions in NCC immunofluorescence and WNK puncta formation following a HK diet. (**A**) Multiplex immunofluorescence of NCC, pNCC, and WNK4 in male and female Dahl SS rats. *n* = 3 per group. Scale bars: 25 μm. (**B**) Quantification of NCC and pNCC signal, average area of WNK bodies, and average WNK body count per mm^2^. *n* = 3 per group, ~10 images captured and analyzed per tissue section. One-way ANOVA was used to compare differences compared with the control (NK). *P < 0.05, **P < 0.01, ****P < 0.0001.

As it was previously reported, the expression of ion channels and transporters could be modulated by dietary interventions. As an example, Dahl SS rats exhibited increased renal K_ir_7.1 expression when dietary K^+^ was increased (56). Thus, we examined the expression of main sodium and potassium channels and transporters located in the aldosterone-sensitive distal nephron (ASDN) from renal cortical tissue to observe how the kidney may have compensated for these dietary-induced physiological changes. Examining the expression of channels and transporters in these groups (Figure 6, A and B) revealed no changes in ROMK, a significant increase in K_ir_4.1 only in DK males, and a significant increase in the expression of K_ir_5.1 in male and female DK rats. The A1 subunit of the Na^+^/K^+^-ATPase increased only in male DK animals. The transporter NCC is regulated by potassium intake and has a consequential role in sodium and potassium homeostasis, so it would be significantly affected by our chronic dietary interventions (31). We performed multiplex immunofluorescence of NCC, phosphorylated NCC (pNCC), and WNK4 (Figure 7A) to examine how these changed in both sexes for each dietary condition. Immunofluorescence NCC and pNCC signals were quantified, which revealed decreased NCC and pNCC intensity in male DK rats (Figure 7B); however, in female rats, NCC was significantly increased in DK and HK groups, and pNCC intensity was decreased in HK female rats. With-no-lysine kinas (WNKs) aggregate with SPAK1/OSR to form WNK bodies dependent on potassium intake and modulate NCC phosphorylation (57, 58). We observed the formation of WNK bodies in DK males and females, indicated by the significantly increased average area of WNK4 puncta (Figure 7B). Interestingly, WNK4 puncta were smaller in HK males but larger in HK females. However, these differences are small compared with profound changes in DK groups. The number of WNK4 puncta was lower in HK males, was not different in HK females, but was significantly higher in DK females. These data indicate compensation of the DCT and collecting duct (CD) to retain potassium as a response to the hypokalemic conditions observed in rats fed the DK diet and indicate a clear sex difference in DCT remodeling between potassium-supplemented males and females.

## Discussion

The Na^+^/K^+^ ratio is a well-established and significant determinant in developing SS hypertension (11). We previously observed that decreasing this ratio through potassium supplementation attenuated the development of SS hypertension in male rats over a period of 5 weeks, which we again showed here in the HK males (45). Curiously, the males that were fed a DK diet were even further protected from the development of SS hypertension, even though the Na^+^/K^+^ ratio is magnitudes higher than that of the NK or HK groups. Furthermore, the DK male rats had the best probability of survival, though they were not healthy, given the severe hypokalemia and lower heart rates. Potassium is essential in the growth and development of muscle tissue, which contains roughly 80% of the body’s intracellular potassium (59, 60). When extracellular K^+^ levels decrease, skeletal muscle can release its intracellular potassium stores to act as a buffer (61). Given more time on the diet, it is likely that the DK males would eventually experience high rates of mortality, likely due to cardiac arrhythmia induced by severe hypokalemia (62). In addition to blood pressure, heart rates were also reduced in the DK group; a cohort study involving over 600 patients utilized cardiovascular magnetic resonance and found an association of left ventricular indexed stroke volume with cardiac remodeling (63). We observed extensive collagen formation in the left ventricular tissue of DK rats, which suggests that their stroke volume was impaired. The DK groups likely had a reduced cardiac output, the product of stroke volume and heart rate, which would contribute significantly to the reduced blood pressure observed. Female DK rats were also extremely hypokalemic, though unlike males, they experienced a high rate of mortality. This may be attributed to the lower muscle mass and resulting stores of potassium in females, which causes lessened tolerance to chronic potassium deficiency. Additionally, potassium supplementation did not lower blood pressure in the female HK group; instead, it trended toward a slight elevation compared with the NK females. Natriuresis was unchanged in male and female HK groups, indicating mechanisms aside from the natriuretic effect of high potassium that participated in the difference in phenotype. Furthermore, we did not observe any renoprotection with potassium supplementation, as there were no differences in cortical fibrosis or medullary protein casts between HK and NK rats of both sexes; although the HK diet significantly attenuated blood pressure in male rats, their pressure was still elevated and was not lowered sufficiently to reduce renal damage. It is well known that females are more protected against hypertension than males, though they are more susceptible to salt sensitivity, but this protection tends to fall off at older age (64, 65). However, an analysis of the EPIC-Norfolk cohort found that even at older age (average of about 60 years old), the blood pressure–reducing effects of potassium were more profound in women than men (66). Additionally, a recent study modeled the effects of sex differences in sodium and potassium intake and suggests that the differences in the abundance of renal transporters in women are a significant factor in attenuated blood pressure compared with that of men (67).

Aldosterone, which increases sodium retention and potassium excretion, was significantly increased in male and female HK rats in line with their excretion of potassium. Interestingly, the female HK group had considerably higher circulating aldosterone levels than male HK rats, which may partly explain why the females who were supplemented with potassium did not experience an attenuation in blood pressure. Sex hormones are known to influence aldosterone production; it has been observed that estrogen binds to and inhibits the angiotensin type 1 receptor (AT_1_R), therefore lowering production of aldosterone (68–70). A postmenopausal decrease in estrogen production is a significant factor in increased rates of hypertension in aging females, and some evidence suggests that hormone replacement therapy can reverse these effects (68, 71). However, a previous study using Sprague-Dawley rats observed that female rats were more sensitive to aldosterone infusion and experienced an increase and decrease in plasma sodium and potassium, respectively (72). Furthermore, estradiol treatment in ovariectomized female rats had adverse effects, resulting in elevated plasma sodium and reduced plasma potassium, but potassium levels returned to normal with dietary supplementation (72). More work is required to unravel the sex differences in the relationship between RAAS and potassium, though it is evident that sex hormones should be taken into consideration when assessing potential therapeutic interventions.

Many studies have previously examined the effects of low dietary potassium in various animal models, though the results have been somewhat inconsistent. Classical experiments by Dahl et al. examined how the Na^+^/K^+^ ratio affected blood pressure development in the SS rat. They observed that the ratio inversely correlated with blood pressure, with the lowest potassium diet containing 0.17% KCl (11). While relatively low, it is not as extreme as our and many other studies. Several studies conducted in mice utilized more severe dietary potassium depletion and saw an increase in blood pressure with low potassium (73–76). Each of these studies also presented a compensatory increase of NCC and pNCC, indicating a remodeling of the DCT to accommodate the low K^+^ load. One study in spontaneously hypertensive rats (SHRs) and another in Long Evans rats implemented severe potassium restriction and reported findings similar to ours, though no renal mechanisms were studied or proposed (77, 78). Conditions of low potassium have been established to augment phosphorylation and activity of NCC (32, 79–81). This mechanism is dubbed the “potassium switch” and is essential for proper sodium and potassium handling in the distal nephron. K_ir_4.1 is involved in this mechanism, and the WNK signaling pathway was shown to modulate NCC in the DCT1 (31, 57, 58, 82). In our experiments, we observed that DK animals exhibited extremely low kaliuresis due to the relative absence of potassium in the diet. K_ir_5.1 was significantly upregulated in male and female DK animals, indicating compensation in the distal nephron for reabsorbing filtered potassium, but K_ir_4.1 was only increased in males. The lack of compensation of K_ir_4.1 in females may have resulted in less reabsorption of potassium and contributed to their increased mortality. Previous work examining sexual dimorphisms discovered that female animals have differing expressions of renal transporters, especially distal sodium transporters, on normal and high-salt diets (43, 44, 83). These dimorphisms in renal transporters and channels may partially explain the blood pressure differences we observed between males and females and their tolerance to high and low potassium loading.

Exploring the transcriptomic profiles of the rat kidneys revealed further insights into the plausible mechanisms involved. The gene *Slc38a3* encoding for the glutamine transporter SNAT3 was significantly upregulated in male and female potassium-deficient groups in the present study. In previous studies done in mice, it was shown that, in K^+^ deficiency with metabolic acidosis, SNAT3 is upregulated, suggesting a role in ammonia-genesis (84). Furthermore, it was also shown that mice chronically supplemented with K^+^ had decreased levels of *Slc38a3* (75). Therefore, potential metabolic acidosis might contribute to the weaker survival rate in rats on DK diet. Interestingly, male rats fed DK and HK diets had lower MAP and *Il6* and *Hmox1* mRNA levels compared with normal K^+^-fed rats. The cytokine *Il6* is increased in many different hypertensive conditions, and a decrease of it has been shown to attenuate hypertension and kidney damage in Dahl SS rats (50, 85, 86). Since *Hmox1* is a cytoprotective enzyme that degrades prooxidant heme, an increase in it is usually associated with a decreased blood pressure phenotype (87). However, *Il6* and *Hmox1* expression are linked (88) and may have caused the decreased levels in the current study. A major insight of the transcriptomic data was the enhanced Ca^2+^ signaling in the DK rats. Lowering dietary potassium was shown to increase intracellular calcium and activate the CREB pathway in a previous study done in mice (89). As dietary potassium regulates vascular calcification, it might have affected the rats that had low mortality in the current study as well.

There are some limitations to our study. A diet that is almost entirely deficient in potassium is not very translatable. While the Western diet is low in potassium, it is not devoid of it. Thus, these findings should not be utilized to make such a comparison. The study was not designed to mimic the Western diet or to study hypokalemia, but rather to examine the compensatory mechanisms and adaptations to such stressful conditions. Additionally, dietary potassium is typically found conjugated to citrate or HCO_3_^–^, while we used KCl for supplementation (90, 91). Although less commonly consumed, KCl salt substitutes are commercially available and may see an increase in use given the proven clinical benefits of salt substitution (17). During the dietary protocol, no food or water measurements were done to record intake. There were no differences in urine volume, so water intake was likely not drastically different between groups. The DK rats exhibited decreased blood pressure and natriuresis; recording food intake would have allowed us to differentiate whether these results are solely due to physiological changes in the animals or partially due to reduced consumption of NaCl. Initial and periodic urine measurements during the protocol would also be insightful to examine the progression and renal adaptation to the dietary challenges. Unfortunately, due to equipment availability constraints, we were only able to collect on the final day and were still unable to do so for every experimental animal. Despite these setbacks, our data indicate a plethora of physiological changes in response to changes in dietary potassium.

Altogether, we demonstrate that a DK diet significantly reduces the K^+^ load, induces hypokalemia, and — contrary to many prior findings — prominently blunts the development of SS hypertension in a translatable animal model. Our data suggest the potassium channels and transporters in the CD play a significant role in compensatory adaptations to chronic hypokalemia and that there are clear sex differences in how dietary potassium affects SS hypertension. Overall, these findings emphasize the complex interplay between dietary K^+^, Na^+^, and sex-specific responses in the regulation of blood pressure and cardiorenal health. The renal control and regulation play crucial roles in hypertension under varying dietary Na^+^/K^+^ conditions, influencing both cardiovascular and cardiorenal outcomes. Understanding these mechanisms is essential for developing targeted dietary and therapeutic interventions for hypertension management.

## Methods

### Sex as a biological variable.

WT male and female Dahl SS rats were used for experimental studies.

### Animals.

Dahl SS rats were used for our experiments; animals were obtained from an inbred colony at the Medical College of Wisconsin (Milwaukee, Wisconsin, USA) and maintained at the University of South Florida. Animals were weaned at 3 weeks of age and maintained on a normal salt (0.4% NaCl; 0.36% K^+^) diet (Dyets Inc.; D113755). Between 8 and 9 weeks of age, animals were implanted with a radio telemeter (PA-C40; Data Sciences International [DSI]), which was threaded through the femoral artery and into the descending aorta. Ponemah software (DSI) was used to record blood pressure. Animal housing and recording rooms were under a 12:12 light/dark cycle in which lights were on at 6:00 a.m. and off at 6:00 p.m. Daily blood pressure and heart rate values are an average of recordings from 9:00 a.m. to 12:00 p.m., during which entering the recording room was prohibited. Following recovery from surgery animals were switched to 1 of 3 high-salt (4% NaCl) diets, a NK (0.36% K^+^) diet (Dyets Inc.; D113756 NK), a HK (1.41% K^+^) diet supplemented with KCl (Dyets Inc; D113522 HK), or a DK (1.57E-06% K^+^) diet containing only trace amounts of potassium (Dyets Inc.; D105028). Rats were maintained on these diets for 5 weeks, and upon reaching the endpoint, animals were anesthetized with isoflurane, the descending aorta was catheterized, and kidneys were flushed with PBS and collected before euthanasia.

### Plasma and urine measurement.

On the final day of the dietary protocol, rats were placed in metabolic cages for 24-hour urine collection. At the endpoint, just before the kidneys were flushed, heparinized blood was collected from the descending aorta, centrifuged at 6,000*g* for 5 minutes, and the separated plasma was collected. Urine and plasma samples were analyzed using a radiometer (ABL800 FLEX, Radiometer America Inc.) to measure the concentrations of K^+^, Na^+^, Cl^–^, Ca^2+^, and creatinine.

### IHC.

The collected kidney tissue was fixed in 10% formalin and embedded in paraffin blocks. Tissue blocks were sliced at 4 μm–thick slices, deparaffinized, and stained with Masson’s trichrome. Stained kidney slides were imaged with an Olympus VS120 slide scanner and blindly analyzed for cortical fibrosis and medullary protein casts. ImageJ software (NIH) was used to color-threshold the respective areas to determine the area percentage of cortical fibrosis or medullary protein casts.

Collected heart tissue was fixed in 10% formalin, embedded in paraffin blocks, and sectioned at 4 μm. PSR staining was performed as explained previously (92). For each animal/slide, 4–6 images of left ventricular tissue were blindly acquired with a KEYENCE BZ-X800 microscope. Using ImageJ software, percentage of stained area was determined and averaged to determine the percent area stained for collagen density in the left ventricle.

### Measurement of plasma RAAS metabolites.

Quantification of RAAS metabolites in plasma samples was performed by the University of Kentucky (Lexington, Kentucky, USA) RAAS analytical lab in partnership with Attoquant Diagnostics by method of liquid chromatography–tandem mass spectrometry as previously described (47, 93).

### RNA-Seq.

Kidney cortical tissue was homogenized, and Trizol reagent was used to extract total RNA following the manufacturer’s recommended protocol (Thermo Fisher Scientific). RNA was quantified and its purity was verified using a BioTek Synergy Neo2 plate reader before being sent for sequencing at Novogene. The raw and processed data provided by Novogene was further analyzed and visualized by the authors.

### Functional analysis and the visualization of transcriptomic data.

To determine the DE genes, *P* < 0.05 was considered significant. Further analyses were done using IPA (QIAGEN). First, core analyses were done for HK and DK groups, each compared with NK within the same sex. Then, comparison analyses were done for both core analyses within each sex. Lists generated from said analyses were used to overlay or predict associated diseases, functional annotations, and canonical pathways. Heatmaps with hierarchical clustering, principal component analysis (PCA) plots, and volcano plots were created using the open-source software R. R packages; ggplot, ggplot2, dplyr, pheatmap, reactlog, circlize, ggrepel, and tidyverse were used in the process.

### Western blotting analysis.

Collected kidney tissue was snap-frozen in liquid nitrogen. The frozen kidney cortex was cut and sonicated in 2× Laemmli buffer containing protease and phosphatase inhibitors. After transfer to a nitrocellulose membrane, the membranes were blocked in Every Blot Blocking Buffer (Bio-Rad, 12010020). Membranes were then incubated in primary antibodies at a concentration of 1:1,000 overnight, followed by HRP-tagged secondary antibodies for an hour. Membranes were exposed to ECL reagents, visualized with a ChemiDoc (Bio-Rad), and analyzed with Image Lab software (Image Lab 6.1, Bio-Rad). Primary antibodies included ROMK (Alomone Labs, APC-001), K_ir_4.1 (Abclonal, A9826), K_ir_5.1 (MilliporeSigma, SAB4501636-100UG), and A1 Na^+^/K^+^-ATPase (Santa Cruz Biotechnology Inc., SC-21712).

### Immunofluorescence and microscopy.

Kidney tissue from paraffin blocks were sectioned at 4 μm, before being deparaffinized and rehydrated. For antigen retrieval, sections were permeabilized in 0.125% SDS for 5 minutes, quenched with buffer (75 mM NH_4_Cl, 100 mM glycine, pH 8.0) for 15 minutes, and blocked in PBS with 2% BSA (Sigma-Aldrich) for 1 hour. FlexAble CoraLite Plus (dye wavelengths, 488 and 647) Antibody Labeling Kits for Rabbit IgG (Proteintech, KFA001 and KFA003) were used for multiplex of 2 rabbit antibodies, NCC (StressMarq, SPC-402) and pNCC (PhosphoSolutions, p1311-53); antibodies were linked with FlexLinker at a 1:1 ratio. Sections were incubated overnight with the multiplexed NCC and pNCC antibodies along with a guinea pig WNK4 antibody at concentrations of 1:150 (57). The WNK4 antibody was provided by Arohan Subramanya, University of Pittsburgh (produced by Pocono Rabbit Farm and Laboratory). Primary antibodies were washed, and sections were incubated with an Alexa Fluor 594–conjugated guinea pig secondary antibody (Invitrogen, A-11076) at 1:1,000 for 1 hour. Slides were then mounted with coverslips. Fluorescence images were captured with an Axio Observer 7 (Zeiss) inverted microscope equipped with an Axiocam 702 camera. Using an oil immersion 40× objective with a 1.3 numerical aperture, about ten 7-step *z* stacks were acquired for each slide. ImageJ software was used to analyze NCC and pNCC intensity and the number and size of WNK bodies. For each image, *z* stacks were summed, and regions of interest (ROIs) were drawn around the apical fluorescent area for NCC and pNCC. The intensity of fluorescence was determined by calculating the mean gray area. For WNK bodies, ROIs were drawn for the entire DCT segments, and WNK bodies were selected by image thresholding. The “analyze particle” plugin was used to calculate the number of WNK bodies and the area of each one.

### Statistics.

Graph Pad Prism 9.3 software (GraphPad Software) was utilized to visualize data and perform statistical analysis. Data were analyzed using 2-way ANOVA or 1-way ANOVA with Dunnett’s multiple-comparison test; the NK group was used as the control mean. Summarized data are reported as mean ± SEM, and statistical significance was determined at *P* < 0.05.

### Study approval.

All animal experiments followed the *Guide for the Care and Use of Laboratory Animals* (National Academies Press, 2011), and all protocols were reviewed and approved by the IACUC at the University of South Florida.

### Data availability.

Data presented in the current work are available in the Supporting Data Values file and upon reasonable request from the corresponding authors. The RNA-Seq datasets are available at: https://www.ncbi.nlm.nih.gov/geo/query/acc.cgi?acc=GSE255582

## Author contributions

Conceptualization was contributed by AZ, AS. Investigation was contributed by AZ, LVD, ML, BX, VL, CAK, VK, and GVH. Analysis was contributed by AZ, LVD, CAK, OP, VK, and GVH. Writing of the original draft was contributed by AZ, LVD, and AS. Review, editing, and final approval were contributed by AZ, LVD, ML, BX, VL, VK, GVH, CAK, OP, and AS. Resources and supervision were contributed by CAK, OP, and AS. AZ and AS are the guarantors of this work and, as such, had full access to all the data in the study and take responsibility for the integrity of the data and the accuracy of the data analysis.

## Supplementary Material

Supplemental data

Supporting data values

## Figures and Tables

**Table 1:**
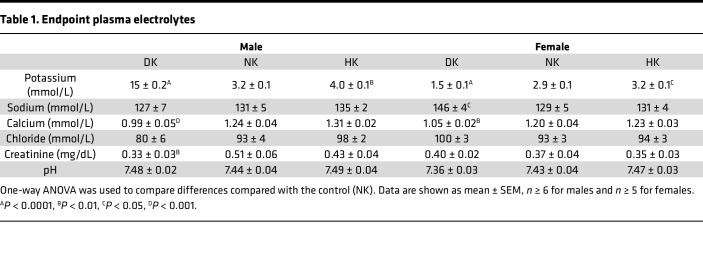
Endpoint plasma electrolytes

**Table 2:**
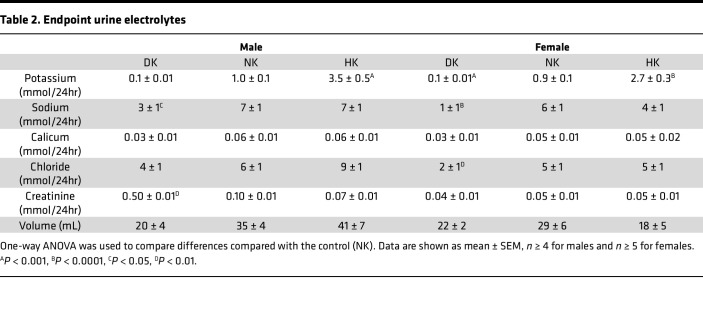
Endpoint urine electrolytes

## Version 1 04/15/2025

In-Press Preview

## Version 2 05/22/2025

Electronic publication
