# Supplementary material for: Potassium supplementation and depletion during development of salt-sensitive hypertension in male and female SS rats
Source: JCI Insight. 2025 Apr 15;10(10):e181778. doi: 10.1172/jci.insight.181778 (PMC12128975; doi:10.1172/jci.insight.181778)
Supplement: Supplemental data [file jciinsight-10-181778-s271.pdf]

SUPPLEMENTAL DATA

Effects of potassium supplementation and depletion on the development of salt-sensitive hypertension in male and female Dahl SS rats

Adrian Zietara, Lashodya V. Dissanayake, Melissa Lowe, Biyang Xu, Vladislav Levchenko, Vasundhara Kain, Ganesh V Halade, Christine A. Klemens, Oleg Palygin, Alexander Staruschenko

Supplemental Figures and Tables:

Supplemental Figure 1. Endpoint mean arterial pressure and heart rate on a HS diet.

Supplemental Figure 2. Systolic and diastolic blood pressure during HS diet.

Supplemental Figure 3. Quantification of plasma RAAS metabolites between males and females.

Supplemental Figure 4. Estimated RAAS measurements.

Supplemental Figure 5. Clustering of RNA sequencing data based on diet and sex.

Supplemental Figure 6. Overall transcriptomic changes in Males.

Supplemental Figure 7. Overall transcriptomic changes in Females.

Supplementary Table 1. Hypertension-related differentially expressed genes in male DK vs. NK comparison.

Supplementary Table 2. Hypertension-related differentially expressed genes in female DK vs. NK comparison.

Supplementary Table 3. Hypertension-related differentially expressed genes in male HK vs. NK comparison.

Supplementary Table 4. Hypertension-related differentially expressed genes in female HK vs. NK comparison.

Supplementary Table 5. Differentially expressed genes that participate in ion transport in male DK vs. NK comparison.

Supplementary Table 6. Differentially expressed genes that participate in ion transport in female DK vs. NK comparison.

Supplementary Table 7. Differentially expressed genes that participate in ion transport in male HK vs. NK comparison.

Supplementary Table 8. Differentially expressed genes that participate in ion transport in female HK vs. NK comparison.

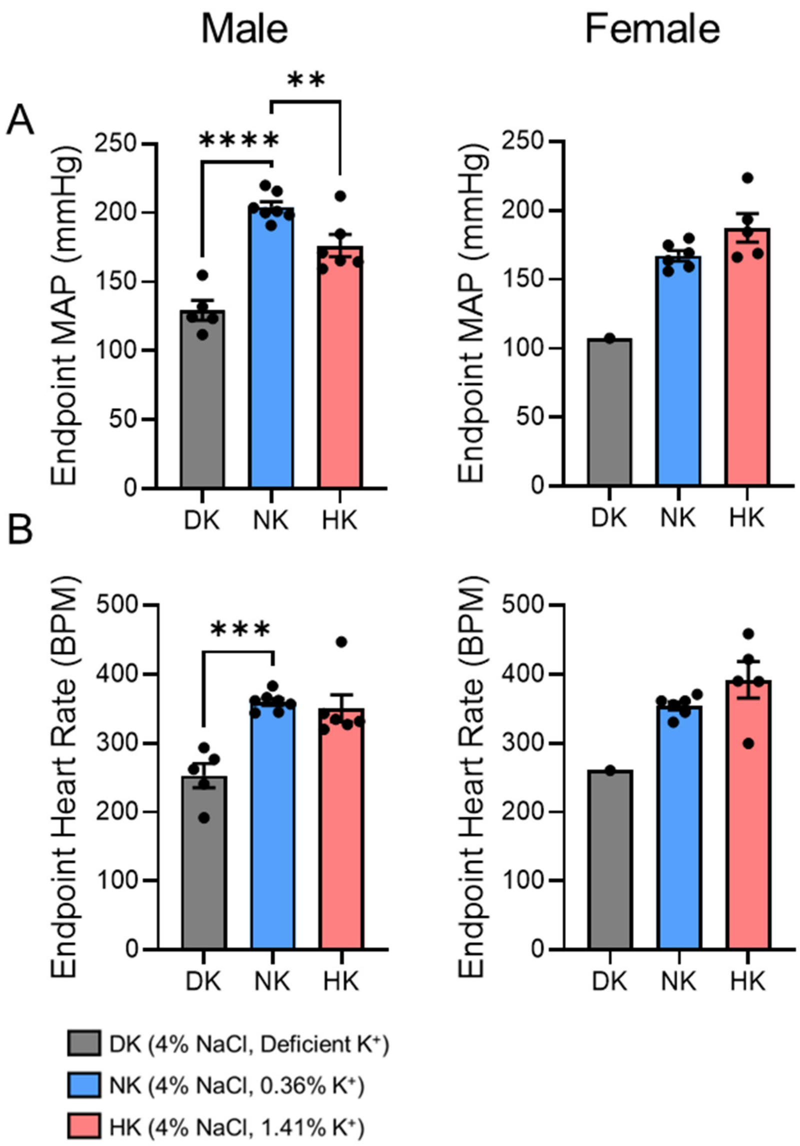

Supplemental Figure 1. Endpoint mean arterial pressure and heart rate on a HS diet. (**A**) Endpoint mean arterial pressure of male and female rats after 5 weeks of 4% NaCl diet. (**B**) Endpoint heart rate of male and female rats after 5 weeks of 4% NaCl diet. One-way ANOVA was used to compare differences compared to the control (NK). Data are shown as mean ± SEM, $N \geq 5$ for males and $N \geq 5$ for females ($N \geq 1$ for female DK group). **$P < 0.01$, ***$P < 0.001$, ****$P < 0.0001$.

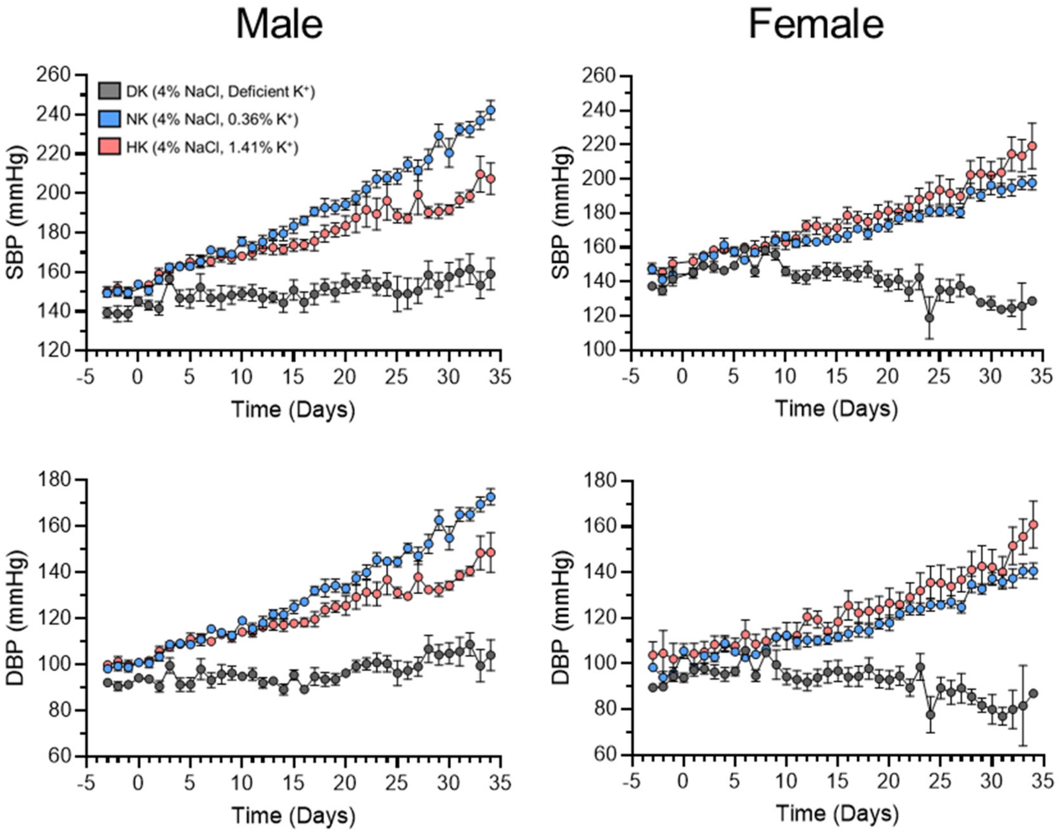

Supplemental Figure 2. Systolic and diastolic blood pressure during HS diet. (**A**) Daily average mean systolic blood pressure (SBP) and (**B**) diastolic blood pressure (DBP) of male and female SS rats during five weeks on a 4% NaCl diet with either deficient (DK), normal (NK), or high (HK) potassium. Daily values are averages of recordings from 9:00 am to 12:00 pm. Two-way repeated measure ANOVA was used to compare differences between groups. Data are shown as mean ± SEM, $N \geq 5$ for males and $N \geq 5$ for females ($N \geq 1$ for female DK group).

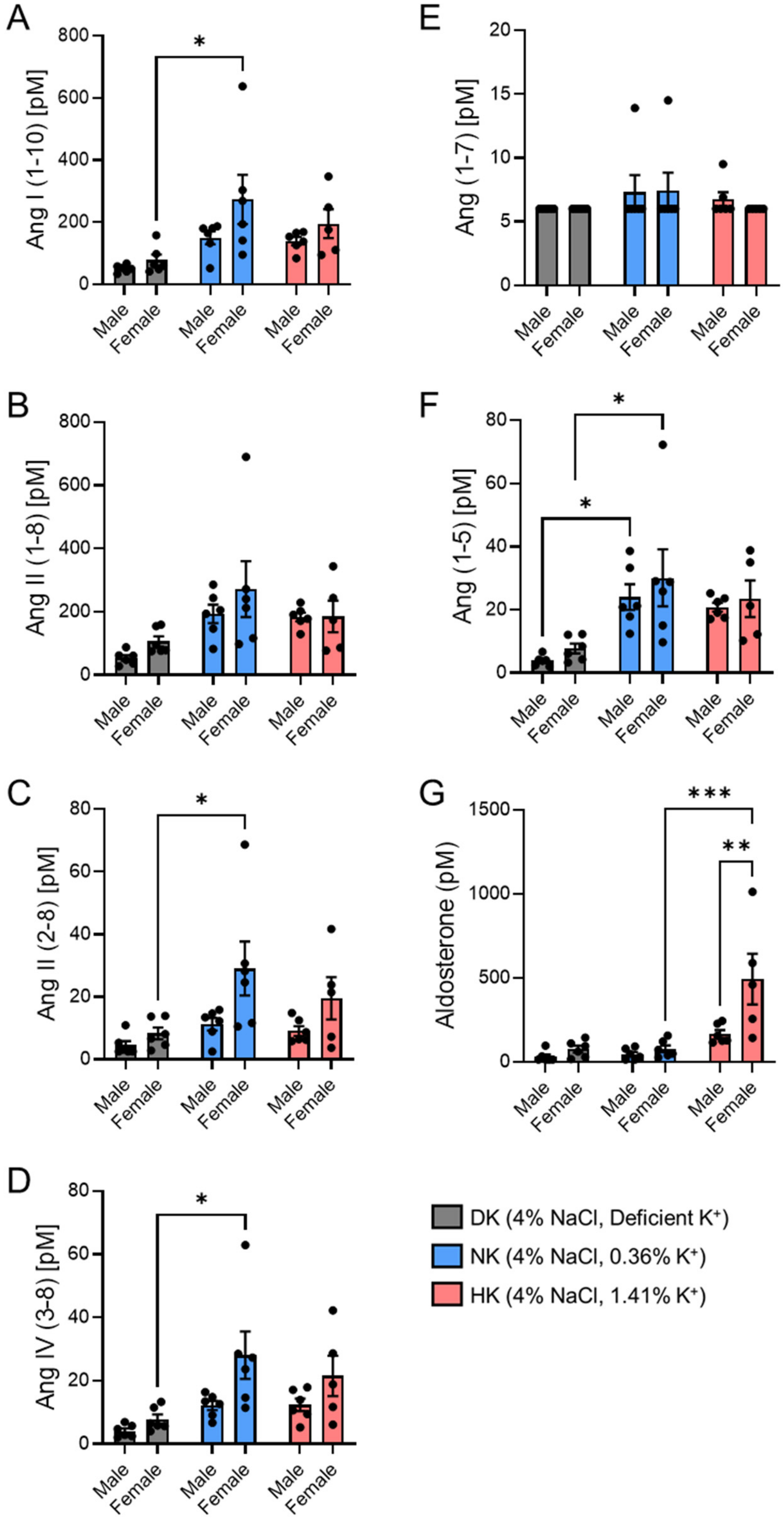

Supplemental Figure 3. Quantification of plasma RAAS metabolites between males and females. (**A**) Male-female comparisons for Ang I (1-10), (**B**) Ang II (1-8), (**C**) Ang III (2-8), (**D**) Ang IV (3-8), (**E**) Ang (1-7), (**F**) Ang (1-5), (**G**) Aldosterone. One-way ANOVA was used to compare differences compared to the control (NK) and two-way ANOVA was used to compare differences between sex and diet. Data are shown as mean ± SEM, $N \geq 6$ for males and $N \geq 5$ for females. *$P < 0.05$, **$P < 0.01$, ***$P < 0.001$, ****$P < 0.0001$.

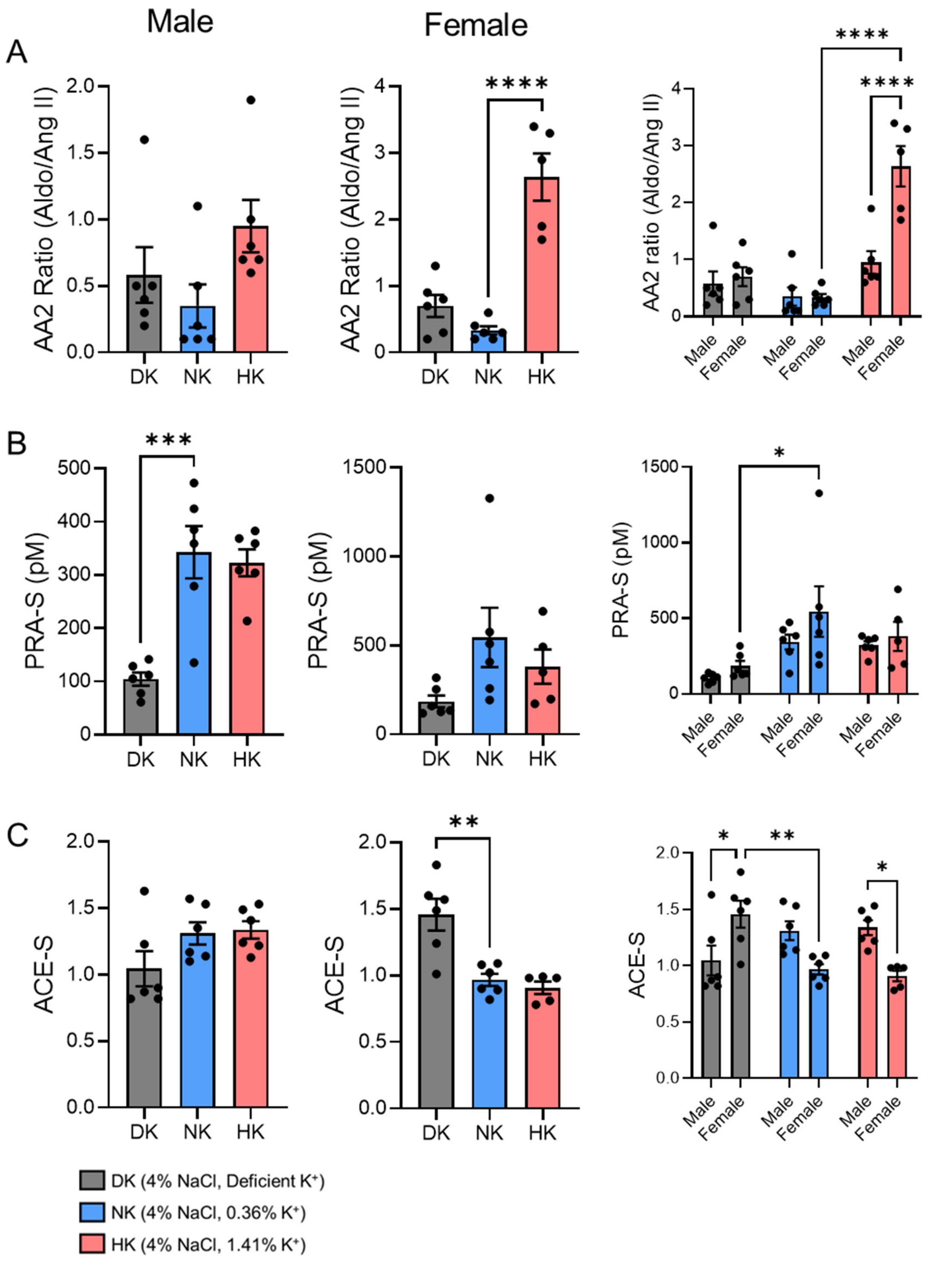

Supplemental Figure 4. RAAS activity surrogates. (**A**) Ration of aldosterone to Ang II (AA2 ratio). (**B**) Estimated plasma renin activity (Ang I + Ang II). (**C**) Estimated angiotensin converting enzyme activity (Ang II/Ang I). Data are shown as mean ± SEM, $N \geq 6$ for males and $N \geq 5$ for females. *$P < 0.05$, **$P < 0.01$, ***$P < 0.001$, ****$P < 0.0001$.

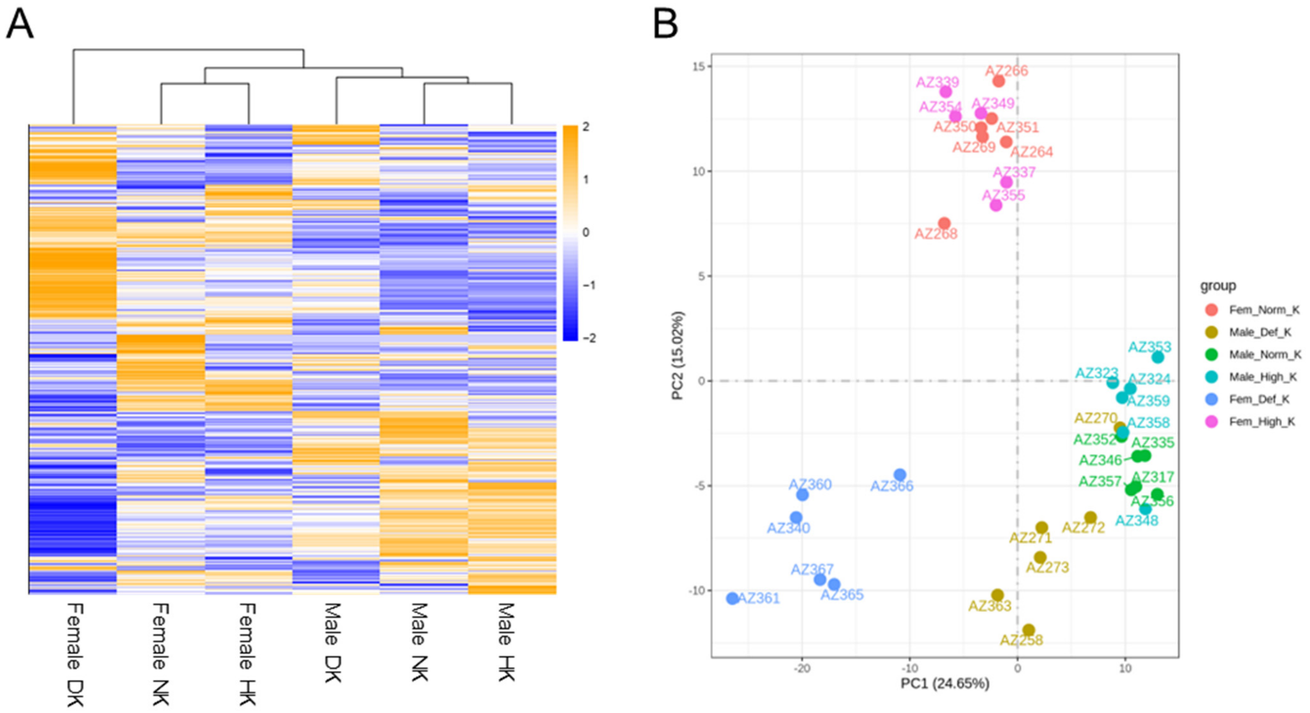

Supplemental Figure 5. Clustering of RNA sequencing data based on diet and sex. (**A**) Hierarchical clustering. (**B**) Principal component analysis (PCA) plot. $N \geq 5$ per group.

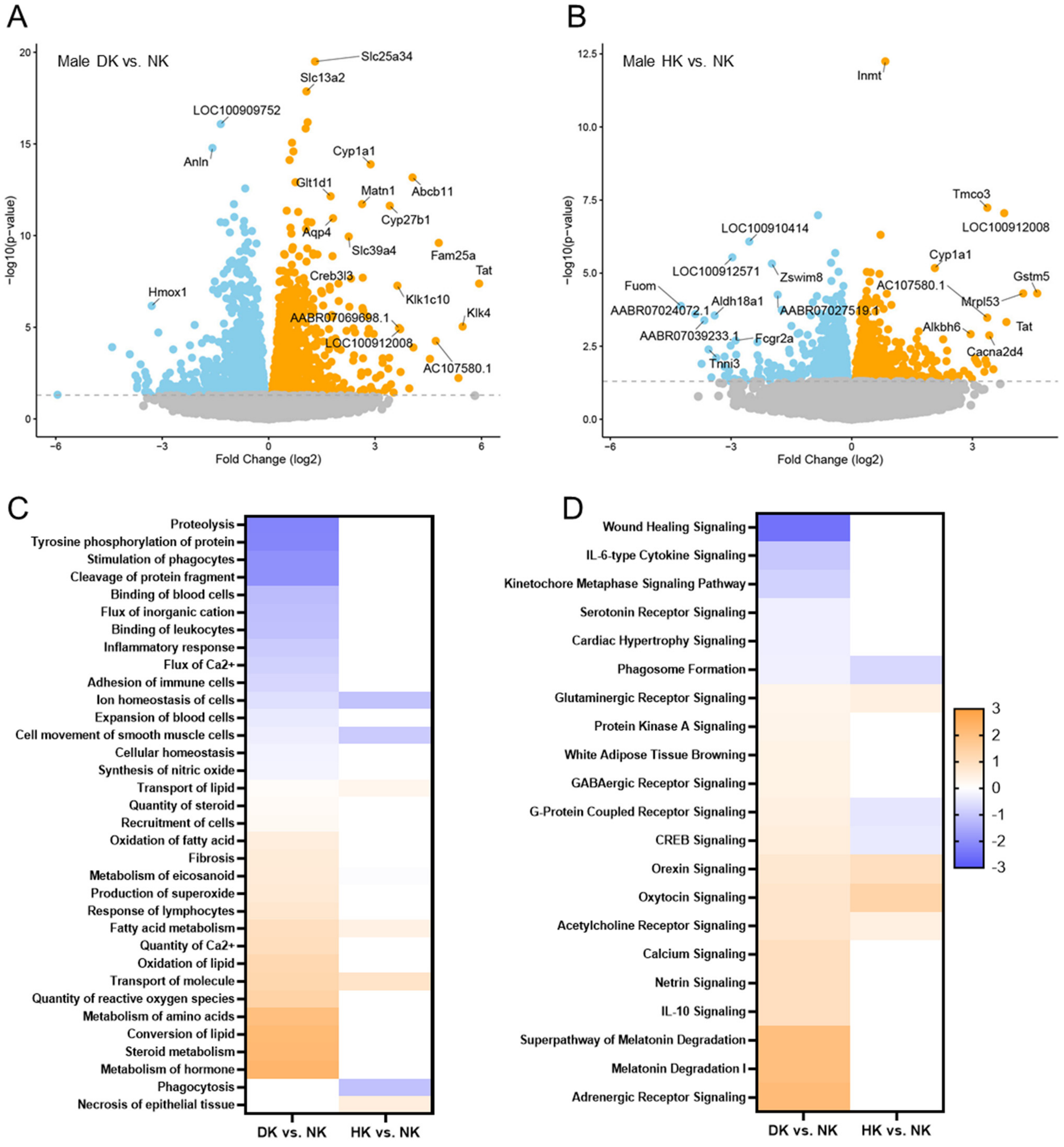

Supplemental Figure 6. Overall transcriptomic changes in Males. (**A**) Volcano plot for male DK vs. NK. (**B**) Volcano plot for male HK vs. NK. (**C**) Top biological function and disease annotations (**D**) Top canonical pathways. *N* ≥ 5 per group.

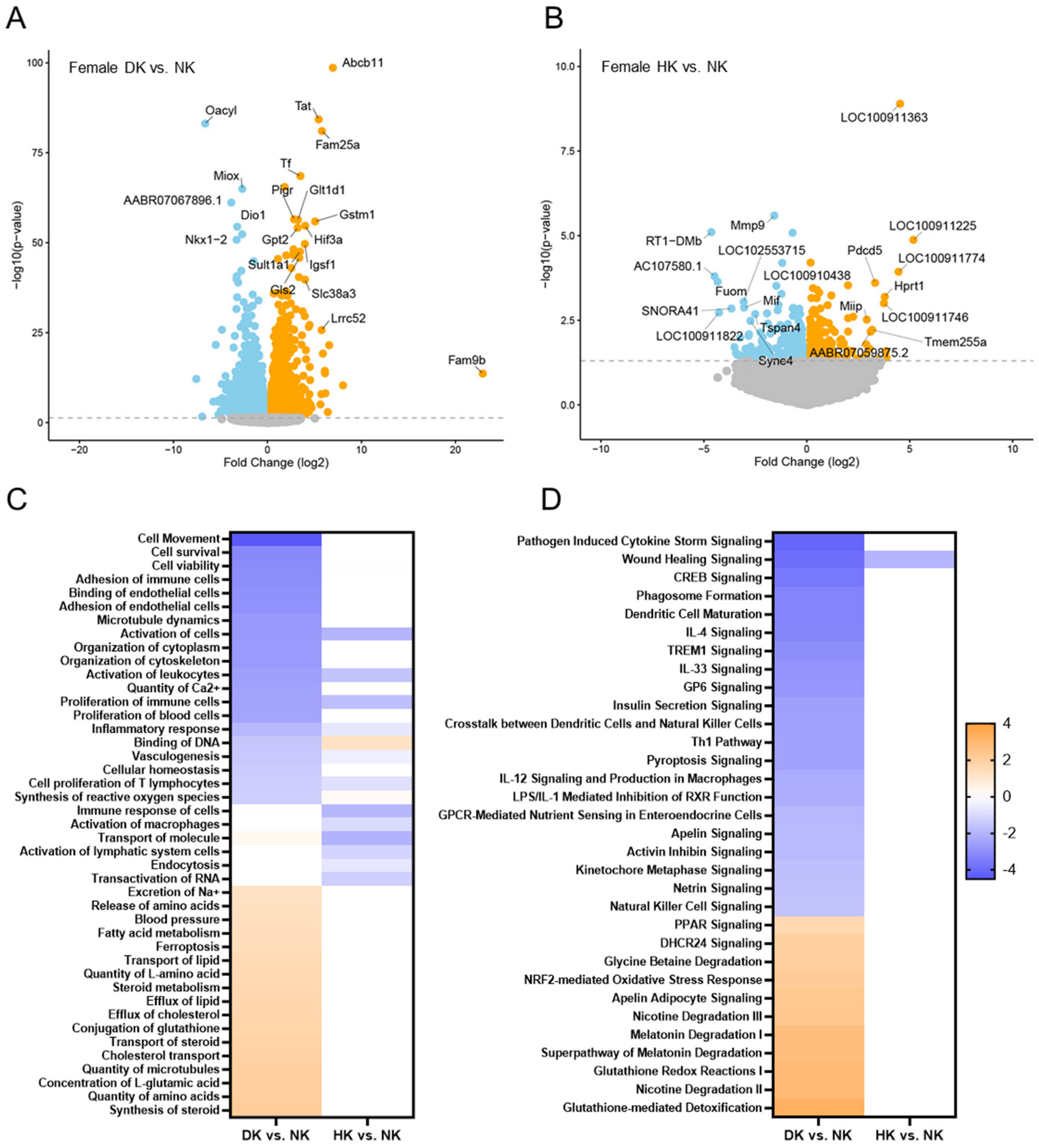

Supplemental Figure 7. Overall transcriptomic changes in Females. (**A**) Volcano plot for male DK vs. NK. (**B**) Volcano plot for male HK vs. NK. (**C**) Top biological function and disease annotations (**D**) Top canonical pathways. *N* ≥ 5 per group

Supplementary Table 1. Hypertension-related differentially expressed genes in male DK vs. NK comparison. Expr Fold change and p-value are data from the current study. Species evidence and causal or correlated columns show where the evidence in literature comes from for the relationship between the gene and “Hypertension”.

Gene Symbol	Molecule Type	Expr Fold Change	Expr p-value	Species Evidence	Causal or correlated
Glud1	enzyme	1.095	1.78E-11	Rat	correlation
Adamts4	peptidase	-1.167	6.92E-09	Human	correlation
Cxcl11	cytokine	1.028	4.12E-08	Human	correlation
Klk1	peptidase	3.628	5.38E-08	Human	correlation
Pde9a	enzyme	1.01	8.96E-08	Human	correlation
Ttk	kinase	-1.432	1.48E-07	Human	correlation
Hmgcs2	enzyme	1.975	3.74E-07	Rat	correlation
Hmox1	enzyme	-3.296	6.81E-07	Human, Mouse, Rat	causal, correlation
Cyp2e1	enzyme	1.26	1.53E-06	Human	correlation
F5	other	1.22	1.82E-06	Human	correlation
Gsta3	enzyme	1.733	2.72E-06	Human	correlation
F2	peptidase	-1.214	2.06E-05	Human	correlation
Cacna2d1	ion channel	-1.202	3.21E-05	Human, Rat	correlation
Gstm5	enzyme	1.291	0.000112	Rat	correlation
Cyp11a1	enzyme	1.056	0.000209	Human	correlation
Cxcl13	cytokine	-1.062	0.000517	Human	correlation
Nptx2	other	-1.495	0.00212	Human	correlation
Tac3	other	-1.42	0.00638	Human	correlation
Zbtb16	transcription regulator	1.064	0.00787	Human	correlation
Ciart	other	1.445	0.00855	Human	correlation
Prkg1	kinase	-1.422	0.0135	Human, Mouse	causal, correlation
Gng7	other	-1.143	0.0149	Human	correlation
Foxf1	transcription regulator	-1.16	0.0159	Human	causal, correlation
Serpina6	other	-1.187	0.0174	Rat	correlation
Scn1a	ion channel	-2.248	0.0177	Human	correlation
Adra2c	G-protein coupled receptor	1.152	0.0186	Human	correlation
Il6	cytokine	-1.276	0.023	Human, Rat	correlation
Tf	transporter	1.074	0.0281	Human	correlation
Mmp10	peptidase	-2.875	0.0303	Human	correlation
F10	peptidase	-1.038	0.0356	Human	correlation
Stox1	other	1.082	0.0395	Human	causal, correlation
Lmntd1	other	-3.091	0.0449	Human	correlation
Il11	cytokine	-1.047	0.0487	Human	correlation

Supplementary Table 2. Hypertension-related differentially expressed genes in female DK vs. NK comparison. Expr Fold change and p-value are data from the current study. Species evidence and causal or correlated columns show where the evidence in literature comes from for the relationship between the gene and "Hypertension".

Gene symbol	Molecule Type	Expr Fold Change	Expr p-value	Species Evidence	Causal or correlated
Tf	transporter	3.502	2.89E-69	Human	correlation
Gstm5	enzyme	5.062	1.25E-56	Rat	correlation
Rarres2	transmembrane receptor	2.764	6.79E-49	Human	correlation
Cyp2e1	enzyme	2.695	2.22E-47	Human	correlation
Gsta3	enzyme	2.537	1.05E-43	Human	correlation
Krt8	other	1.985	2.26E-33	Rat	correlation
Glud1	enzyme	2.023	4.10E-30	Rat	correlation
Enpp3	enzyme	1.797	6.73E-27	Rat	correlation
Mmp9	peptidase	-4.841	1.62E-26	Human	correlation
Aspa	enzyme	-1.596	8.71E-25	Rat	correlation
Mthfr	enzyme	-2.429	3.84E-22	Human	correlation
Esr1	ligand-dependent nuclear receptor	-1.822	6.87E-21	Human	correlation
Ccl2	cytokine	-2	2.59E-19	Mouse	correlation
Cyp11a1	enzyme	1.834	3.63E-18	Human	correlation
Gbp2	enzyme	-1.095	1.28E-16	Human, Rat	correlation
Thbd	transmembrane receptor	-1.152	2.69E-16	Human	correlation
Adra2b	G-protein coupled receptor	1.582	2.78E-16	Human	correlation
Cd14	transmembrane receptor	1.289	3.58E-16	Rat	causal
Otog	enzyme	-1.666	1.58E-15	Human	correlation
F5	other	1.619	1.74E-15	Human	correlation
Cxcl13	cytokine	-3.405	7.07E-15	Human	correlation
Gstp1	enzyme	2	7.23E-15	Human	correlation
Tgm2	enzyme	-1.94	9.65E-15	Human	correlation
Nqo1	enzyme	1.682	1.14E-14	Rat	correlation
Asgr1	transmembrane receptor	2.26	1.51E-14	Human	correlation
Pcdh12	other	-1.282	1.71E-14	Human	correlation
Scg2	cytokine	-2.435	4.24E-14	Human	correlation
Asic2	ion channel	-2.27	6.54E-14	Human	correlation
Cxcl12	cytokine	-1.553	9.91E-14	Human	correlation
Chrnb1	transmembrane receptor	1.062	1.00E-12	Human	correlation
Idh1	enzyme	-1.014	5.03E-12	Rat	correlation
Oas1	enzyme	-1.839	6.24E-12	Rat	correlation
Cxcl11	cytokine	1.531	7.08E-12	Human	correlation
Angptl2	other	-1.611	8.51E-12	Human	correlation

Acox1	enzyme	1.015	9.70E-12	Human	correlation
Lonrf3	other	1.701	1.31E-11	Human	correlation
Sema5b	other	-1.883	1.31E-11	Human	correlation
Pde9a	enzyme	1.245	2.07E-11	Human	correlation
Phgdh	enzyme	-1.226	4.72E-11	Rat	correlation
Ace2	peptidase	-1.45	1.87E-10	Human, Mouse	causal, correlation
Slc16a12	transporter	-1.044	3.15E-10	Human	correlation
Kl	enzyme	-1.324	5.98E-10	Mouse, Human, Rat	causal, correlation
Lgals1	other	-1.109	1.21E-09	Human	correlation
Kcnip1	ion channel	-1.355	1.68E-09	Human	causal
Neto2	other	-1.487	1.92E-09	Human	correlation
Kap	other	-1.737	2.45E-09	Mouse	causal
Acox2	enzyme	-1.55	2.53E-09	Human	correlation
Vwf	other	-1.589	4.07E-09	Human	correlation
Fbln5	other	-1.219	5.35E-09	Rat	correlation
Edn1	cytokine	1.492	6.38E-09	Human, Mouse, Rat	causal, correlation
Slc39a8	transporter	-1.008	7.75E-09	Human	correlation
Fibin	other	1.592	9E-09	Human	correlation
Slc6a4	transporter	-1.362	1.26E-08	Mouse, Human	causal, correlation
C1qb	other	1.025	1.6E-08	Human	correlation
St6gal1	enzyme	-1.325	2.04E-08	Human	correlation
Nadsyn1	enzyme	1.003	2.13E-08	Human	correlation
Oxtr	G-protein coupled receptor	-2.743	2.65E-08	Human	correlation
Hmgcs2	enzyme	2.894	4.05E-08	Rat	correlation
Ca6	enzyme	1.151	4.7E-08	Human	correlation
Ndufa4l2	enzyme	-2.221	4.79E-08	Human	correlation
Nos1	enzyme	-1.666	6.3E-08	Mouse, Rat	causal, correlation
G6pd	enzyme	1.012	6.39E-08	Rat	causal
Aplnr	G-protein coupled receptor	-1.16	6.44E-08	Rat	correlation
Slit3	other	-1.232	8.77E-08	Human	correlation
Cacna1h	ion channel	-1.198	9.4E-08	Human	correlation
Egfr	kinase	1.063	1.39E-07	Human	correlation
Tet2	enzyme	-1.296	2.47E-07	Human	correlation
Dbp	transcription regulator	-1.438	2.74E-07	Human	correlation
Fap	peptidase	-2.372	3.67E-07	Human	correlation
Serpina6	other	-4.752	3.73E-07	Rat	correlation
Igsf10	other	-1.282	3.88E-07	Human	correlation
Ca7	enzyme	-1.145	3.95E-07	Human	correlation
Ahr	ligand-dependent nuclear receptor	-1.07	5.26E-07	Mouse, Rat	causal
Gcm1	transcription regulator	-1.893	6.82E-07	Human	correlation
Adora2b	G-protein coupled receptor	1.162	8.51E-07	Mouse	causal

Pik3cg	kinase	-1.187	9.28E-07	Human, Mouse	causal, correlation
Mrc1	transmembrane receptor	1.264	1.1E-06	Human, Rat	correlation
Inmt	enzyme	1.311	1.8E-06	Human	correlation
C6	other	1.076	2.14E-06	Human	correlation
Notch3	transcription regulator	-1.146	2.23E-06	Human	correlation
Ren	peptidase	1.62	3.91E-06	Human, Mouse, Rat	causal, correlation
Gdf15	growth factor	1.89	4.32E-06	Human	correlation
Tac3	other	-4.697	5.11E-06	Human	correlation
Ptpru	phosphatase	1.124	5.23E-06	Rat	correlation
Cacna1c	ion channel	-1.492	8.29E-06	Human, Rat	correlation
Abcc9	ion channel	-1.031	1.06E-05	Mouse	causal
Eln	other	-1.062	1.14E-05	Mouse	causal
Chrna2	transmembrane receptor	-2.126	1.21E-05	Human	correlation
Ptgs2	enzyme	-1.615	1.57E-05	Human, Mouse	causal, correlation
Spon1	other	-1.303	1.99E-05	Human	correlation
St8sia4	enzyme	-1.515	0.00003	Human	correlation
Guca2b	other	1.432	4.24E-05	Mouse	causal
Tlr9	transmembrane receptor	-1.335	5.66E-05	Human	correlation
Skil	transcription regulator	-1.164	5.87E-05	Human	correlation
Angpt2	growth factor	-1.292	6.06E-05	Mouse	causal
Ccl4	cytokine	-1.761	0.000064	Human	correlation
Ciart	other	-1.829	7.91E-05	Human	correlation
Piga	enzyme	1.097	9.04E-05	Human	correlation
Cd40	transmembrane receptor	-1.122	9.71E-05	Human	correlation
Hmox1	enzyme	2.883	0.000106	Human, Mouse, Rat	causal, correlation
Tnnc2	other	2.487	0.000107	Human	correlation
Akap12	transporter	1.202	0.000196	Human	correlation
Snca	enzyme	-3.934	0.000217	Human	correlation
F10	peptidase	-1.574	0.000247	Human	correlation
Prrx1	transcription regulator	-1.02	0.000356	Human	correlation
Kcnh1	ion channel	-1.975	0.00039	Human	correlation
Tmem119	other	-1.296	0.000556	Human	correlation
Bche	enzyme	-1.77	0.000603	Human	correlation
Krt23	other	1.146	0.00093	Human	correlation
Sntb1	other	-1.043	0.000932	Human	correlation
Slc14a1	transporter	-1.874	0.001	Rat	correlation
Klrd1	transmembrane receptor	-1.077	0.00107	Human	correlation
Aff3	transcription regulator	-1.441	0.00111	Human	correlation
Thbs2	other	-1.021	0.00117	Human	correlation

Ankrd55	transcription regulator	1.627	0.00166	Human	correlation
Chrne	transmembrane receptor	1.073	0.00178	Human	correlation
Notum	enzyme	3.628	0.00206	Human	correlation
Actg2	other	-2.391	0.00211	Human	correlation
Kcna4	ion channel	-2.814	0.00216	Human	correlation
Trpc4	ion channel	-2.691	0.00264	Human	correlation
Ttk	kinase	-1.745	0.00273	Human	correlation
Pcsk6	peptidase	-1.684	0.0034	Human, Mouse	causal, correlation
Zbtb16	transcription regulator	1.045	0.00448	Human	correlation
Dok6	other	-3.58	0.00554	Human	correlation
Dpysl3	enzyme	-1.18	0.00733	Human	correlation
Epha3	kinase	-3.078	0.00763	Human	correlation
Mmp7	peptidase	1.649	0.00771	Human, Mouse, Rat	causal, correlation
Cdc7	kinase	-1.02	0.00778	Human	correlation
Gabre	ion channel	1.557	0.00986	Human	correlation
Slc24a2	transporter	2.952	0.01	Human	correlation
Add2	other	-3.316	0.0116	Mouse	causal
Foxf1	transcription regulator	-1.248	0.0142	Human	causal, correlation
Gzma	peptidase	-1.7	0.0143	Human	correlation
Cacna2d1	ion channel	-1.025	0.0144	Human, Rat	correlation
Prkg1	kinase	-1.388	0.0144	Human, Mouse	causal, correlation
Asb4	transcription regulator	-2.521	0.0176	Mouse	causal
Vgf	growth factor	3.199	0.0229	Mouse	causal
Gabrb3	ion channel	-1.294	0.0275	Human	correlation
Slc7a11	transporter	-2.152	0.0279	Human	correlation
Asic4	ion channel	-1.397	0.0301	Human	correlation
Alb	transporter	-1.513	0.0321	Human	correlation
Glp1r	G-protein coupled receptor	-1.333	0.0349	Human	correlation
Nptx2	other	-1.087	0.0359	Human	correlation
Robo4	other	-2.995	0.0399	Human	correlation
Mmp10	peptidase	-3.566	0.0412	Human	correlation
Trem1	transmembrane receptor	2.013	0.0412	Human	correlation
Nppa	other	3.124	0.0466	Human, Mouse	causal, correlation
Calcoco1	transcription regulator	1.106	0.0491	Human	correlation

Supplementary Table 3. Hypertension-related differentially expressed genes in male HK vs. NK comparison. Expr Fold change and p-value are data from the current study. Species evidence and causal or correlated columns show where the evidence in literature comes from for the relationship between the gene and Hypertension.

Gene Symbol	Molecule Type	Expr Fold Change	Expr p-value	Species Evidence	Causal or correlated
Hmox1	enzyme	-2.071	0.0115	Human, Mouse, Rat	causal, correlation
Slc14a2	transporter	3.357	0.013	Mouse	causal
Il6	cytokine	-1.245	0.0145	Human, Rat	correlation
Oxtr	G-protein coupled receptor	1.014	0.021	Human	correlation
Mir-192	microRNA	2.789	0.022	Human, Rat	causal, correlation
Mmp3	peptidase	-2.691	0.0248	Human	correlation
Ciart	other	1.054	0.0391	Human	correlation
Chrnb2	transmembrane receptor	1.325	0.0412	Human	correlation
Ms4a1	other	-1.468	0.0454	Human	correlation
Dsc3	other	-2.031	0.048	Human	correlation

Supplementary Table 4. Hypertension-related differentially expressed genes in female HK vs. NK comparison. Expr Fold change and p-value are data from the current study. Species evidence and causal or correlated columns show where the evidence in literature comes from for the relationship between the gene and Hypertension.

Gene Symbol	Molecule Type	Expr Fold Change	Expr p-value	Species Evidence	Causal or correlated
Alb	transporter	-2.228	0.0327	Human	correlation
Ciart	other	-1.166	0.0404	Human	correlation
Hmgcs2	enzyme	1.461	0.0477	Rat	correlation
Hnrnpc	other	-1.889	0.00397	Uncategorized	correlation
Hoxa4	transcription regulator	-2.146	0.01	Human	correlation
Mif	cytokine	-3.045	0.00134	Human, Rat	causal, correlation
Mmp9	peptidase	-1.578	2.55E-06	Human	correlation
Pparg	ligand-dependent nuclear receptor	-1.172	0.0423	Human, Mouse, Rat	causal, correlation
Robo4	other	-3.44	0.0295	Human	correlation
Snca	enzyme	-1.364	0.0489	Human	correlation

Supplementary Table 5. Differentially expressed genes that participate in ion transport in male DK vs. NK comparison.

Gene symbol	Expr Fold Change	Expr p-value	Molecule Type
Cyp27b1	3.42	2.35E-12	enzyme
Slc39a4	2.26	1.13E-10	transporter
Tfrc	1.09	5.03E-10	transporter
Klk1	3.63	5.38E-08	peptidase
F2	-1.22	2.06E-05	peptidase
Cacna2d1	-1.21	3.21E-05	ion channel
Slc25a25	1.29	8.99E-05	transporter
Slc38a3	1.7	0.00016	transporter
Calb1	1.12	0.001029	other
Cntn1	-1.05	0.001304	enzyme
Lrrc52	3.38	0.0018	ion channel
Slc13a4	1.4	0.002313	transporter
Kcnk4	1.45	0.003055	ion channel
Hcn4	-1.84	0.004761	ion channel
Sfrp4	-1.75	0.008265	transmembrane receptor
Scn1a	-2.25	0.017692	ion channel
Tf	1.08	0.028148	transporter
Ucp1	-5.95	0.047943	transporter
Cacna2d4	2.68	0.048325	ion channel

Supplementary Table 6. Differentially expressed genes that participate in ion transport in female DK vs. NK comparison. Expr Fold change and p-value are data from the current study.

Gene symbol	Expr Fold Change	Expr p-value	Molecule Type
Tf	3.51	2.89E-69	transporter
Slc38a3	3.99	1.83E-40	transporter
Slc39a4	2.45	1.61E-30	transporter
Lrrc52	5.76	1.89E-26	ion channel
Slc15a3	-1.29	3.92E-21	transporter
Trpv1	-1.39	7.5E-19	ion channel
Cyp27b1	4.11	2.49E-18	enzyme
Slc22a1	-1.53	1.93E-16	transporter
Trpm1	-1.71	2.12E-15	ion channel
Slc44a5	-2.61	5.02E-14	transporter
Asic2	-2.27	6.54E-14	ion channel
Cxcl12	-1.56	9.91E-14	cytokine
Slc25a25	3.01	3.43E-13	transporter
Cyp2c23	1.18	6.15E-13	enzyme
Chrnb1	1.07	1E-12	transmembrane receptor
Slc5a6	-1.31	4.78E-12	transporter
Scara5	2.4	6.76E-12	transmembrane receptor
Kcne1b	-2.57	8.16E-12	ion channel
Slc4a5	-2.26	9.43E-12	transporter
F2rl1	1.1	6.29E-11	G-protein coupled receptor
Slc39a14	1.14	2.52E-10	transporter
Slc13a4	2.03	3.33E-10	transporter
Kl	-1.33	5.98E-10	enzyme
Slc5a12	1.13	7.81E-10	transporter
Cldn10	-1.11	1.33E-09	other
Panx1	1.18	1.93E-09	transporter
Dpp6	-1.52	4.65E-09	other
Cp	1.24	5.79E-09	enzyme
Edn1	1.5	6.38E-09	cytokine
Ccr5	-1.11	7.47E-09	G-protein coupled receptor
Slc39a8	-1.01	7.75E-09	transporter
Gja5	-1.22	7.85E-09	transporter
Clca4	1.1	1.63E-08	ion channel
Slc8a2	-2.23	2.47E-08	transporter
Slc17a3	1.19	3.13E-08	transporter
Cldn16	-1.11	3.73E-08	transporter
Hcn2	1.02	4.61E-08	ion channel
Tfrc	1.06	5.31E-08	transporter
Nos1	-1.67	6.3E-08	enzyme
Cacna1h	-1.2	9.4E-08	ion channel
Il1b	-1.58	1.66E-07	cytokine
Lrp2	-1.07	2.04E-07	transporter
Slc5a8	1.64	2.68E-07	transporter
Slc15a2	-1.01	5.1E-07	transporter
Slc15a1	1.3	8.24E-07	transporter
Cnr1	-1.22	9.06E-07	G-protein coupled receptor
Abcc2	1.12	9.32E-07	transporter

Kcnj12	-1.04	9.94E-07	ion channel
Atp2c2	-1.75	1.23E-06	enzyme
Cacna1i	-3.33	1.38E-06	ion channel
Trpm2	-1.09	1.7E-06	ion channel
Trpv5	1.08	4.66E-06	ion channel
Cacna1c	-1.5	8.29E-06	ion channel
Abcc9	-1.04	1.06E-05	ion channel
Bhlha15	2.49	2.09E-05	transcription regulator
Kcnmb1	-1.81	2.22E-05	ion channel
Tg	-1.91	2.22E-05	other
Trpv3	-1.27	3.93E-05	ion channel
Piezo2	-1.27	5.62E-05	ion channel
Slc41a2	-1.05	7.65E-05	transporter
Kcnk2	1.11	0.000132	ion channel
Gjd3	-1.46	0.000154	transporter
Cd22	-1.07	0.000183	transmembrane receptor
Slc24a3	-1.82	0.000277	transporter
Kcnh1	-1.98	0.00039	ion channel
Slc26a2	-1.24	0.000409	transporter
Scn8a	-1.09	0.000488	ion channel
Slc22a7	-1.1	0.000553	transporter
Edn3	-1.67	0.001059	other
Kcnip2	6.42	0.001233	transporter
Chrne	1.08	0.001775	transmembrane receptor
Kcna4	-2.82	0.002165	ion channel
Trpm3	-1.38	0.002416	ion channel
Kcnmb4	1.27	0.002587	ion channel
Trpc4	-2.7	0.002636	ion channel
Cftr	-1.42	0.004243	ion channel
Kel	1.68	0.004327	peptidase
Trpv6	1.07	0.00476	ion channel
Ano3	2.29	0.005433	transporter
Slc12a5	-1.25	0.00598	transporter
Clca1	3.69	0.006887	ion channel
Slc26a9	-1.16	0.007264	transporter
Slco1a1	-3.4	0.007502	transporter
Meltf	1.7	0.008334	other
Catsper4	2.6	0.009358	transporter
Gabre	1.56	0.009864	ion channel
Slc24a2	2.96	0.010037	transporter
Add2	-3.32	0.011626	other
Cacna2d1	-1.03	0.014368	ion channel
Slc1a7	-1.2	0.016314	transporter
Cntn1	-1.81	0.019031	enzyme
Cysltr1	-1.31	0.020712	G-protein coupled receptor
Aqp8	3.33	0.023488	transporter
Kcnn2	-1.02	0.024426	ion channel
Trpa1	-1.11	0.026207	transporter
Gabrb3	-1.3	0.027531	ion channel
Kcnk16	-1.01	0.031863	ion channel
Nmur1	1.02	0.040052	G-protein coupled receptor

Gnas	-1.14	0.04418	enzyme
Nppa	3.13	0.046582	other

Supplementary Table 7. Differentially expressed genes that participate in ion transport in male HK vs. NK comparison.

Gene symbol	Expr Fold Change	Expr p-value	Molecule Type
Cacna2d4	3.42	0.001346	ion channel
Sfrp4	-1.56	0.00412	transmembrane receptor
Slc1a7	1.1	0.010261	transporter
Trpm5	1.31	0.018144	ion channel
Ano5	-3.05	0.022544	ion channel
Kcng4	-3.15	0.032218	ion channel
Best3	1.12	0.03323	ion channel
Cacna1i	-1.77	0.040318	ion channel
Chrnb2	1.33	0.041243	transmembrane receptor
Ano3	-3.02	0.049393	transporter

Supplementary Table 8. Differentially expressed genes that participate in ion transport in female HK vs. NK comparison.

Gene symbol	Expr Fold Change	Expr p-value	Molecule Type
Mif	-3.05	0.001338	cytokine
Slc12a5	-1.12	0.007276	transporter
Slc24a3	-1.2	0.015303	transporter
Kcnk7	-2.01	0.017242	ion channel
Clca1	2.89	0.033947	ion channel
Trpm5	1.5	0.035436	ion channel
Pparg	-1.18	0.042301	ligand-dependent nuclear receptor
